# Whether the Indications for Reverse Shoulder Arthroplasty Should Continue to Be Expanded? A Systematic Review and Meta‐Analysis

**DOI:** 10.1111/os.14311

**Published:** 2024-12-12

**Authors:** Huankun Li, Hangsheng Bao, Zhidong Yang, Baijun Hu, Yaocheng Pan, Yi Wang, Jiayi Chen, Hongjun Chen, Bisheng Shen, Yonggen Zou

**Affiliations:** ^1^ The Eighth Clinical Medical College of Guangzhou University of Chinese Medicine Foshan China; ^2^ Zhongshan Hospital of Traditional Chinese Medicine Affiliated to Guangzhou University of Traditional Chinese Medicine Zhongshan China

**Keywords:** anatomical total shoulder arthroplasty, hemi‐arthroplasty, indications, meta‐analysis, reverse shoulder arthroplasty

## Abstract

**Background:**

It is still unclear whether reverse total shoulder arthroplasty (RTSA) has advantages over traditional hemiarthroplasty (HA) and anatomic total shoulder arthroplasty (ATSA) in the treatment of complex shoulder joint diseases. Therefore, this study aims to evaluate the clinical effectiveness of RTSA in the treatment of complex shoulder joint diseases and further determine whether it is necessary to expand the indications of RTSA.

**Method:**

We conducted a systematic search of studies published between January 1, 2012 and May 31, 2023 in PubMed, Embase, and Cochrane databases. The experimental group included patients who underwent primary reverse total shoulder arthroplasty (RTSA), while the control group consisted of patients who underwent primary hemiarthroplasty (HA) or anatomic total shoulder arthroplasty (ATSA). The minimum follow‐up period was 1 year, and a random‐effects model was utilized for data synthesis.

**Results:**

A total of 45 studies were included in the meta‐analysis. Compare to HA, RTSA showed significant advantages in postoperative ASES scores (*p* = 0.004), forward flexion (*p* < 0.0001), and abduction (*p* < 0.0001). Compare to ATSA, RTSA showed significantly lower postoperative Constant scores (*p* = 0.004), ASES scores (*p* = 0.001), SST scores (*p* < 0.0001), forward flexion (*p* < 0.0001), abduction (*p* = 0.011), internal rotation (*p* < 0.0001), and external rotation (*p* < 0.0001). Further meta regression analysis was conducted, considering factors such as region, age, gender ratio, and follow‐up time, excluding the influence of relevant factors. Overall, RTSA did not demonstrate advantages in postoperative functional scores and range of motion. In terms of complication and revision rates, RTSA had lower rates compared to HA and ATSA, except for the complication rate, where there was no significant difference between RTSA and ATSA (*p* = 0.521), but statistically significant differences were observed in other measures.

**Conclusion:**

RTSA demonstrates better clinical efficacy compared to HA but is inferior to ATSA. It can be considered for expanding treatment options for elderly patients with 3 or 4‐part proximal humeral fractures, but it is not suitable for treating end‐stage shoulder arthritis and humeral head necrosis. Overall, the decision to use RTSA should be carefully evaluated based on the extent of the patient's rotator cuff injury.

## Graphical abstract

We systematically collect the clinical outcomes of RTSA compared to traditional hemi/anatomical total shoulder arthroplasty (HA/ATSA) and explore whether there it is necessary to further expand the indications for RTSA through meta‐analysis these outcomes.

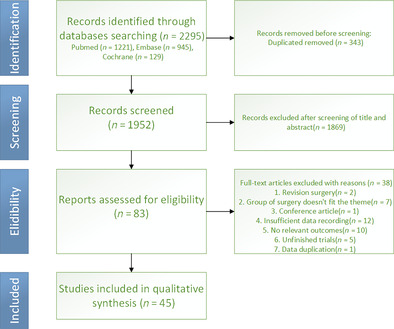

## 1 Introduction

In recent years, the use of reverse shoulder arthroplasty (RTSA) has significantly increased, nearly doubling over the past decade [1, 2], with a continued upward trend expected. Initially indicated for irreparable massive rotator cuff tears [3], RTSA now also addresses complex shoulder conditions, including proximal humeral fractures, humeral head necrosis, shoulder arthritis, and revision surgeries. In cases of comminuted proximal humeral fractures classified as Neer types 3–4, hemi‐arthroplasty (HA) is often performed when open reduction and internal fixation are difficult. However, these fractures frequently involve rotator cuff injuries, complicating healing of the greater tuberosity and repair of the rotator cuff, which can hinder satisfactory outcomes with HA [4, 5]. RTSA overcomes this challenge by shifting the center of rotation inward, enhancing deltoid muscle utilization and reducing reliance on the rotator cuff and the greater tuberosity [6]. This technique is increasingly replacing hemi‐arthroplasty for managing complex proximal humeral fractures.

Anatomical total shoulder replacement (ATSA) was previously considered the preferred method for treating end‐stage shoulder osteoarthritis and humeral head necrosis caused by various factors. However, its postoperative functional recovery heavily relies on the function of the rotator cuff. Patients with such conditions often have severely damaged rotator cuffs due to repeated wear and tear, which can impede postoperative function recovery and increase the risk of failure. In contrast, RTSA overcomes this issue. Numerous studies have now confirmed the clinical effectiveness of RTSA for end‐stage shoulder arthritis and humeral head necrosis [6, 7, 8, 9, 10]. The widespread use and favorable postoperative outcomes of RTSA suggest it may be a superior option for various complex shoulder disorders. However, further research is needed to confirm its clinical efficacy compared to traditional HA and ATSA.

Recent meta‐analyses on shoulder arthroplasty have investigated the treatment of proximal humeral fractures and irreparable rotator cuff injuries, analyzing the clinical efficacy differences among various prostheses and implantation techniques [11, 12, 13]. However, these comparative data are not based on the treatment of the same condition, which does not necessarily indicate that RTSA has a distinct advantage. Over the past decade, the technological maturity of RTSA and improvements in prosthetic designs have led to more widespread application, and its clinical outcomes have become increasingly objective and reliable. Therefore, this meta‐analysis selects clinical research data comparing RTSA to traditional HA and ATSA published between January 1, 2012, and May 31, 2023. The existing clinical efficacy data will provide a reference for evaluating whether the indications for RTSA should continue to be expanded.

## 2 Methods

### 2.1 Search Strategy

This systematic review adhered to the Preferred Reporting Items for Systematic Reviews and Meta‐Analyses (PRISMA) guidelines and was registered with the international prospective register of systematic reviews (PROSPERO protocol ID CRD42023433103). We conducted searches in PubMed, Embase, and Cochrane databases for clinical studies comparing reverse shoulder arthroplasty with either hemiarthroplasty or anatomical total shoulder arthroplasty from January 2012 to May 2023. An example of our search strategy in the PubMed database is as follows: (“Reverse shoulder arthroplasty” [Mesh] OR reverse total shoulder arthroplasty [Text Word] OR reverse shoulder replacement) AND (Hemiarthroplasty OR shoulder [Text Word] OR arthroplasty [Text Word] OR anatomical shoulder arthroplasty OR anatomical total shoulder arthroplasty [Text Word] OR anatomical shoulder replacement) AND (cuff tear arthropathy OR rotator cuff tear OR rheumatoid arthritis OR avascular necrosis OR proximal humeral fracture).

### 2.2 Inclusion and Exclusion Criteria

The inclusion criteria for this review were: (1) clinical studies utilizing reverse total shoulder arthroplasty, hemiarthroplasty, or anatomical total shoulder arthroplasty for complex shoulder conditions; (2) a minimum follow‐up of 1 year; (3) outcome measures including at least one of the following: functional scores (VAS, Constant, ASES, UCLA, SST), postoperative range of motion, reoperation rate, and complication rate.

The exclusion criteria were: (1) studies that did not include reverse shoulder arthroplasty; (2) non‐clinical studies such as biomechanics, necropsy, reviews, conference abstracts, and unfinished trial protocols; (3) studies with incomplete data; (4) for overlapping data across multiple studies, the most recent or most comprehensive studies were selected; (5) for studies from national databases, duplication was minimized by using the latest publications or conducting a more thorough results analysis.

### 2.3 Data Screening and Extraction

Retrieved citations were managed using Endnote software (version X9.1; Clarivate, Philadelphia, PA, USA). Two researchers independently reviewed the abstracts and citations for initial screening based on the inclusion and exclusion criteria. Full‐text assessments were conducted for potentially eligible articles identified in this process. Discrepancies were resolved through consultation with a third researcher. Additionally, relevant systematic reviews and meta‐analyses were assessed to identify any studies potentially overlooked in the original search.

Data was extracted and organized using Microsoft Excel (version 2019; Microsoft, Redmond, WA), independently conducted by two researchers. The extracted data encompassed basic study information (first author and publication year), study characteristics (experimental design, sample size, male‐to‐female ratio, and follow‐up duration), surgical details (surgical approach, prosthesis type, and postoperative rehabilitation protocol), and clinical outcomes (postoperative functional scores, range of motion, reoperation rate, and complications). Different data characteristics and potential measurement variations among studies were categorized to ensure comparability.

### 2.4 Quality Evaluation

Two researchers independently evaluated the methodological quality of the included studies. Non‐randomized controlled trials were assessed using the Methodological Index for Non‐Randomized Studies (MINORS) scale, while randomized controlled trials were evaluated using the Jadad scale. Discrepancies were resolved through discussions with a third researcher. Higher scores on these scales indicate a lower risk of bias.

### 2.5 Statistical Analysis

Statistical analysis of dichotomous and continuous variables was conducted using Stata/SE 16 software (STATA‐Corp, College Station, TX, USA). Dichotomous outcomes included postoperative reoperation and complication rates, while continuous outcomes comprised postoperative functional scores (VAS, Constant, ASES, UCLA, SST) and range of motion (anterior flexion, abduction, internal rotation, external rotation). When both active and passive range of motion were reported, only active range of motion was included. Given the varied indications for reverse total shoulder arthroplasty (RTSA) compared to traditional hemiarthroplasty (HA) or anatomical total shoulder arthroplasty (ATSA), significant heterogeneity among studies was expected, leading to the use of random‐effects models for analysis a priori. Subgroup analysis comparing RTSA and ATSA was performed based on the similarity of treated diseases involving rotator cuff injuries, categorizing them into “Same disease” and “Different disease” subgroups. Heterogeneity was evaluated using Cochrane's *Q*‐test and *I* ^2^ statistics, with *I* ^2^ values of 25%, 50%, and 75% indicating low, moderate, and high heterogeneity, respectively [14]. In cases of significant heterogeneity (*I* ^2^ > 50%), meta‐regression was conducted to identify potential sources of heterogeneity. Publication bias was evaluated using the Egger test.

## 3 Results

### 3.1 Literature Search Results and Quality Evaluation

**FIGURE 1:**
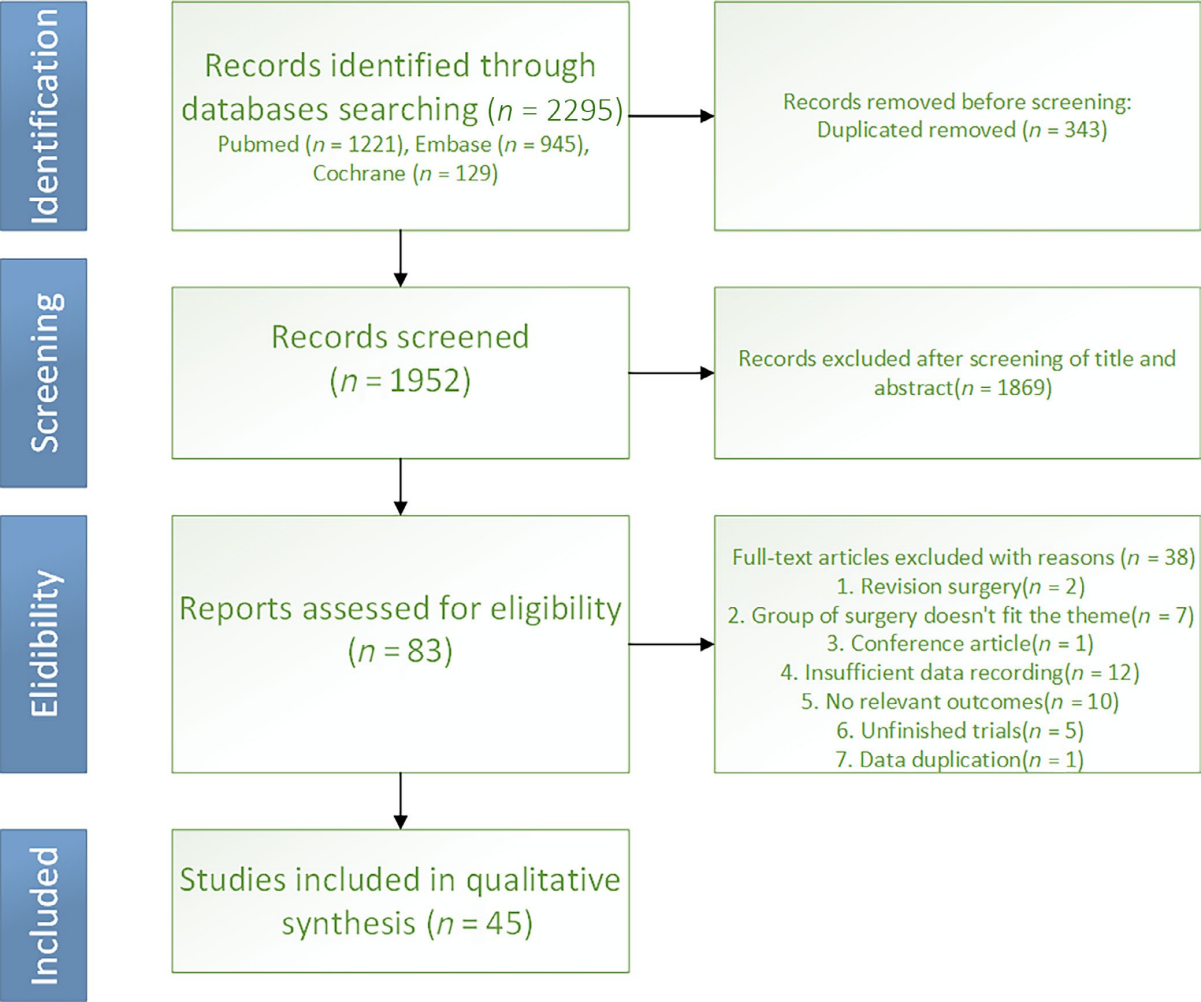
PRISAM (Preferred Reporting Items for Systematic Meta‐analysis) flow diagram.

A total of 2295 citations were retrieved from PubMed, Embase, and the Cochrane Library. After applying inclusion and exclusion criteria, 45 studies were included for quantitative data analysis [4, 7, 8, 9, 10, 15, 16, 17, 18, 19, 20, 21, 22, 23, 24, 25, 26, 27, 28, 29, 30, 31, 32, 33, 34, 35, 36, 37, 38, 39, 40, 41, 42, 43, 44, 45, 46, 47, 48, 49, 50, 51, 52, 53, 54]. The search and screening process is shown in Figure 1. Among the included literature, there were 35 retrospective cohort studies [7, 9, 10, 15, 16, 17, 19, 20, 21, 24, 25, 26, 27, 28, 30, 31, 32, 33, 37, 38, 39, 40, 41, 42, 43, 44, 45, 46, 47, 48, 49, 51, 52, 53, 54] with level III evidence, scoring an average of 16.6 ± 1.6 points on the MINORS scale. Three prospective cohort studies [4, 22, 35] with level II evidence had respective MINORS scores of 17, 16, and 16 points. Four retrospective case–control studies [8, 18, 23, 36] were identified, with one study [18] having level IV evidence and a MINORS score of 15 points. The remaining case–control studies had level III evidence, scoring 18, 16, and 14 points on the MINORS scale, respectively. Three randomized controlled trials (RCTs) [29, 34, 50] were included, all of which were clinical comparative studies on RTSA and HA. One study had level II evidence, while the others had level I evidence, and the Jadad score was 6 points, indicating high quality as shown in Table 1.

**TABLE 1:** Characteristics of included studies.

Study	Study design, level	Time of inclusion, region	Surgical of control group	Age (year)		Population (male/female)		Follow‐time (mouth)		MINORS score
				RSA	Control	RSA	Control	RSA	Control	
Batar 2020 [16]	RCS, III	NR, Turkey	HA	73 ± 6.4	66 ± 7.8	33 (12/21)	25 (7/18)	27 ± 6.1	52 ± 15.4	14
Baudi 2014 [17]	RCS, III	Jan 2008 to Dec 2012, Italy	HA	77	70	25	28	27	26	18
Bonnevialle 2016 [19]	RCS, III	Jan 2009 to Dec 2011, France	HA	78 ± 5	67 ± 10.1	41 (4/36)	57 (18/39)	39 ± 10.1	39 ± 11.6	16
Boyer 2017 [4]	PCS, II	2009–2011, France	HA	78 (66–91)	68 (50–90)	65	69	15 (6–41)	25 (6–96)	17
Chalmers 2014 [20]	RCS, III	NR, USA	HA	77 ± 6	72 ± 7	9	9	14.4	58.8	13
Cuff 2013 [22]	PCS, II	Sep 2007 to Mar 2010, Italy	HA	74.8 (70–86)	74.1 (70–88)	24 (10/14)	23 (9/14)	29 (24–36)	39 (36–48)	16
Han 2020 [27]	RCS, III	Jan 2006 to Jan 2019, China	HA	63.46 ± 8.77	63.37 ± 8.21	64 (31/33)	62 (28/34)	69.24 ± 8.74	69.22 ± 8.81	18
Jonsson 2021 [29]	RCT, I	Sep 2013 to May 2016, Sweden	HA	80.4 ± 4.5	78.6 ± 4.8	41 (2/39)	43 (6/37)	28.8		6^c^
Kany 2021^a^, ^b^ [30]	RCS, III	NR, France	HA	45 (40–50)	CrCo: 43 (29–47); PYC: 44 (35–50)	11 (7/4)	CrCo: 10 (7/3); PYC: 24 (16/8)	53 (24–87)	CrCo: 92 (24–163); PYC: 36 (24–77)	14
Kleim 2021^b^ [33]	RCS, III	May 2013 to Jun 2015, Germany	HA	74.1 (65–84)	58.3 (22–84)	32 (6/26)	21 (17/4)	34.3 (23–55)	26.7 (23–38)	16
Laas 2021 [34]	RCT, II	Oct 2010 to Dec 2015, Netherlands	HA	74.8 (65–92)	75.0 (68–85)	17 (8/9)	14 (5/9)	12		6^c^
Leung 2012 [36]	CCS, III	1997–2007, USA	HA	72 (61–80)	64 (40–82)	20 (8/10)	36 (12/20)	3 (2–5) years	4.4 (2–12) years	14
Repetto 2017 [47]	RCS, III	Jan 2007 to Dec 2011, Italy	HA	71.2 ± 7.5	67.5 ± 10.2	27	24	41.7 ± 17.1	42.2 ± 18.7	16
Sebastia‐Forcada 2014 [50]	RCT, I	2009–2011, USA	HA	74.7 (70–85)	73.3 (70–83)	31 (4/27)	30 (5/25)	29.4 (24–44)	27.7 (24–49)	6^c^
Solomon 2016 [52]	RCS, III	Jan 2007 to Aug 2011, USA	HA	77 (65–88)	77 (68–96)	16 (3/13)	8 (1/7)	43 (18–73)		17
Yahuaca 2020 [54]	RCS, III	1999–2018, USA	HA	73 ± 9	65 ± 12	106 (19/87)	108 (31/77)	29 ± 26	21 ± 22	16
Aibinder 2019 [15]	RCS, III	Jan 2010 to Dec 2013, USA	ATSA	68.2 (31–90)		65	35	3.8 (3–8.3) years		18
Baumgarten 2018 [18]	CCS, IV	Collect data began in Dec 2008, USA	ATSA	74 (51–92)	68.9 (42–87)	42 (19/22)	80 (32/42)	44.4 (24–82.8)		15
Cox 2018 [21]	RCS, III	2004–2015, USA	ATSA	71.6 ± 7.9	70.1 ± 7.7	19 (8/11)	19 (8/11)	38.1 ± 31.9	63.1 ± 34.4	17
Cox 2019^a^ [7]	RCS, III	2004–2015, USA	ATSA	73.4 ± 5.8	71.2 ± 5.5	26 (3/10)	52 (6/20)	First: 84.1 ± 22.3; Second: 62.7 ± 28.1	First: 86.8 ± 19.4; Second: 63.4 ± 15.8	17
Erickson 2022 [23]	CCS, III	2015–2019, USA	ATSA	68 ± 7	65 ± 8	154 (64/88)	155 (51/104)	≥ 24		16
Flurin 2015 [24]	RCS, III	NR, France and USA	ATSA	71.8 ± 8.0	66.2 ± 9.0	617 (225/392)	528 (245/283)	37.1 ± 15.1	42.7 ± 21.9	18
Flynn 2020 [25]	PCS, III	NR, USA	ATSA	72.5 ± 6.4	67.0 ± 8.8	139 (43/96)	191 (88/103)	67.8 ± 10.5	73.8 ± 16.3	16
Garcia 2023 [26]	RCS, III	NR, USA	ATSA	75.3 ± 7.76	68.9 ± 9.9	43 (12/31)	43 (17/26)	46.9 ± 23.6	56.7 ± 33.3	14
Hao 2023^a^ [8]	CCS, III	2001–2021, USA	ATSA	72.8 ± 8.0	66.9 ± 7.4	261 (105/156)	771 (357/414)	42.6 ± 24.5	61.6 ± 35.9	18
			ATSA	72.7 ± 8.6	66.7 ± 8.6	87 (35/52)	257 (119/138)	41.8 ± 21.1	66.2 ± 43.9	
Haritinian 2020 [28]	RCS, III	May 2013 to Dec 2015, France	ATSA	71 ± 11	68 ± 7.5	12 (4/8)	39 (11/28)	> 24		16
Kany 2021^b^ [30]	RCS, III	NR, France	ATSA	45 (40–50)	44 (31–50)	11 (7/4)	77 (58/19)	53 (24–87)	95 (24–301)	14
Kiet 2015 [31]	RCS, III	2008–2011, USA	ATSA	NR		53	47	> 24		16
Kirsch 2022 [32]	RCS, III	2015–2018, USA	ATSA	67 ± 3.5	67 ± 4.7	67 (26/41)	67 (28/39)	27.2 ± 6.0	32.8 ± 13.4	18
Kleim 2021^b^ [33]	RCS, III	May 2013 to Jun 2015, Germany	ATSA	74.1 (65–84)	70.0 (58–84)	32 (6/26)	23 (8/15)	34.3 (23–55)	31.6 (23–51)	16
Lawrence 2019 [35]	PCS, II	May 2014 to Jun 2015, USA	ATSA	71.4 ± 8.8	65.7 ± 9.6	64 (23/41)	64 (42/22)	24		16
Loew 2023 [37]	RCS, III	Jan 2013 to Dec 2019, Germany	ATSA	73 (49–92)	66 (34–85)	117 (35/82)	162 (75/87)	24		19
Luthringer 2022 [38]	RCS, III	2007–2020, USA	ATSA	67.8 ± 9.8	64.2 ± 7.2	83 (25/53)	17 (3/14)	44.7	66	18
Magosch 2017 [9]	RCS, III	2006–2013, Germany	ATSA	70.9 ± 7.7	64.9 ± 9.7	33 (13/20)	86 (47/39)	40.2 ± 18.3	48.6 ± 27.6	18
McLaughlin 2022^a^ [39]	RCS, III	Aug 2005 to Aug 2017, multinational database	ATSA	73 ± 7	59 ± 11	67 (19/48)	52 (21/31)	47 ± 27	54 ± 36	18
			ATSA	74 ± 7	59 ± 10	67 (19/48)	52 (22/30)	42 ± 21	53 ± 32	
Merolla 2020 [40]	RCS, III	Jul 2014 to Nov 2016, Italy	ATSA	74 (69–75)	70 (68–72)	32	26	28.5 ± 4.5	29 ± 1.3	14
Mowbary 2022 [41]	RCS, III	2000–2018, New Zealand	ATSA	NR		4681	3499	60		16
Nazzal, E. M. 2023^a^ [10]	RCS, III	2015–2020, USA	ATSA	71.1 ± 6.9	66.0 ± 6.2	24 (6/18)	93 (58/35)	16.2	17.7	16
			ATSA	72.4 ± 8.7		69 (43/26)		19.7		
Parada 2021 [42]	RCS, III	NR, international database	ATSA	72 ± 8	66 ± 9	4158 (1478/2680)	2224 (1090/1134)	22	34	16
Polisetty 2021 [43]	RCS, III	2007–2018, USA	ATSA	74 ± 7.1	73 ± 5.8	63 (25/38)	252 (100/152)	38 (24–85)	54 (24–122)	18
Polisetty 2023 [44]	RCS, III	2007–2019, USA	ATSA	72 ± 6.0	71 ± 6.3	101 (54/47)	101 (54/47)	3 (24–85)	47 (24–122)	18
Poondla 2020^a^ [45]	RCS, III	2004–2018, USA	ATSA	62.9 ± 5.6	62.3 ± 5.1	81 (43/38)	274 (182/92)	3.1 ± 1.3 years	3.3 ± 1.3 years	19
			ATSA	76.3 ± 4.5	75.3 ± 4.6	104 (39/65)	208 (108/100)	2.8 ± 1.2 years	3.3 ± 1.3 years	
Postacchini 2015 [46]	RCS, III	NR, Italy	ATSA	73 ± 1.9	70 ± 4.4	12 (4/8)	12 (4/8)	24		17
Schaller 2022 [48]	RCS, III	Jan 2011 to Jul 2015, UK	ATSA	76.3 (70–90)	74.1 (70–88)	31	44	12		19
Schoch 2022 [49]	RCS, III	Nov 2004 to Aug 2019, multinational database	ATSA	72.0 ± 7.8	66.3 ± 8.7	1881 (921/955)	2706 (998/1699)	46.6 ± 24.9	60.2 ± 35.0	18
Simovitch 2017 [51]	RCS, III	Feb 2002 to Mar 2014, USA	ATSA	72.2 ± 7.7	66.8 ± 8.5	678 (241/437)	505 (217/288)	36.9 ± 15.9	42.0 ± 22.1	16
Triplet 2015 [53]	RCS, III	NR, USA	ATSA	82.6	83.4	33 (13/19)	18 (8/10)	39 (24–87)		14

Abbreviations: CCS, case–control study; PCS, prospective cohort study; RCS, retrospective cohort study; RCT, randomized controlled trial.

^a^ There were different subgroups in the study, among which Cox 2019 was grouped according to dominant hand or not, and the other grouping methods were shown in the baseline data section of the table.

^b^ The study included data on RTSA compared with HA and ATSA.

^c^ Jadad score.

### 3.2 Patient Characteristics

This study included a total of 45 studies [4, 7, 8, 9, 10, 15, 16, 17, 18, 19, 20, 21, 22, 23, 24, 25, 26, 27, 28, 29, 30, 31, 32, 33, 34, 35, 36, 37, 38, 39, 40, 41, 42, 43, 44, 45, 46, 47, 48, 49, 50, 51, 52, 53, 54]. Among them, 16 studies compared RTSA versus HA [4, 16, 17, 19, 20, 22, 27, 29, 30, 33, 34, 36, 47, 50, 52, 54], with 562 patients receiving RTSA (mean age 45.0–80.4 years, mean follow‐up 12.0–69.2 months) and 591 patients receiving HA (mean age 43.0–78.6 years, mean follow‐up 12–92 months). Out of the 16 studies, 12 recorded [16, 19, 22, 27, 29, 30, 33, 34, 36, 50, 52, 54] the number of male and female patients, which included 114 men (26.3%) and 319 women (73.7%) in the RTSA group, and 162 men (35.4%) and 295 women (64.6%) in the HA group. Significant differences were observed between the two groups in terms of sex ratio, mean age, follow‐up time, and disease treatment in each study. In addition, 31 studies compared RTSA versus ATSA [7, 8, 9, 10, 15, 18, 21, 23, 24, 25, 26, 28, 30, 31, 32, 33, 35, 37, 38, 39, 40, 41, 42, 43, 44, 45, 46, 48, 49, 51, 53], with 14,007 patients receiving RTSA (mean age 45–76.3 years, mean follow‐up 12–84.1 months) and 12,779 patients receiving ATSA (mean age 44–75.3 years, mean follow‐up 12–95 months). Among the 31 studies, 26 recorded [7, 8, 9, 10, 18, 21, 23, 25, 26, 28, 30, 32, 33, 35, 37, 38, 39, 42, 43, 44, 45, 46, 49, 51, 53, 55] the number of male and female patients, which included 3558 men (39%) and 5560 women (61%) in the RTSA group, and 4057 men (44.6%) and 5030 women (55.4%) in the ATSA group. Generally, there were notable differences in sex ratio, mean age, follow‐up time, and disease treatment between the two groups in each study as shown in Tables 1 and 2.

**TABLE 2:** Diseases associated with RTSA compared to control treatments.

Study	Surgical of control group	Disease	
		RSA	Control
Batar 2020 [16]	HA	Neer type 3/4 PHF: 7/26	Neer type 3/4 PHF: 11/14
Baudi 2014 [17]	HA	Four‐part PHF with varus/valgus fracture/humeral head dislocation: 5/13/7	Four‐part PHF with varus/valgus fracture/humeral head dislocation: 11/9/8
Bonnevialle 2016 [19]	HA	Four‐part PHF	
Boyer 2017 [4]	HA	Three and four‐part PHF	
Chalmers 2014 [20]	HA	Three and four‐part PHF	
Cuff 2013 [22]	HA	Three‐part/four‐part PHF: 16/8	Three‐part/four‐part PHF: 9/14
Han 2020 [27]	HA	Failed plate osteosynthesis of PHF with Screw cut‐out/humeral head necrosis/Glenoid destruction: 25/16/22	Failed plate osteosynthesis of PHF with Screw cut‐out/humeral head necrosis/Glenoid destruction: 22/12/28
Jonsson 2021 [29]	HA	Three‐part/four‐part PHF: 20/21	Three‐part/four‐part PHF: 24/19
Kany 20210^a^, ^b^ [30]	HA	POA/IA/other: 3/2/6	POA/IA/other: 4/3/3 (CrCo); 12/10/2 (PYC)
Kleim 20210^b^ [33]	HA	POA/RCTA/IRCT: 6/25/1	POA/Fracture sequelae/AVN/IA: 14/1/3/3
Laas 2021 [34]	HA	Three or four‐part PHF with different RC quality (good/not good/not available): 9/3/5	Three or four‐part PHF with different RC quality (good/not good/not available): 10/3/1
Leung 2012 [36]	HA	RCTA	
Repetto 2017 [47]	HA	Three or four‐part PHF	
Sebastia‐Forcada 2014 [50]	HA	Three‐part/four‐part PHF: 5/26	Three‐part/four‐part PHF: 4/26
Solomon 2016 [52]	HA	Complex PHF	
Yahuaca 2020 [54]	HA	Neer type2/3/4 PHF: 1/28/74; unclear: 3	Neer type2/3/4 PHF: 2/30/60; unclear: 16
Aibinder 2019 [15]	ATSA	RCTA/POA with IRCT/AVN/PTA/IFA: 33/25/2/1/4	POA/AVN/PTA: 32/1/2
Baumgarten 2018 [18]	ATSA	RCTA	POA
Cox 2018 [21]	ATSA	RCTA/PHF/OOA: 13/5/1	POA/OOA: 17/2
Cox 20190^a^ [7]	ATSA	RCTA	POA
Erickson 2022 [23]	ATSA	AVN/POA/IA/PHF/RC tear/RCTA/unclear: 1/53/2/3/28/62/5	AVN/POA/IA/RCTA/unclear: 1/147/2/1/4
Flurin 2015 [24]	ATSA	RCTA, RC tear and POA	POA
Flynn 2020 [25]	ATSA	RCTA/RC tear/POA/IFA/AVN/acute fractures/malunion/PTA: 83/14/17/8/7/4/2/4	POA/PTA/IFA
Garcia 2023 [26]	ATSA	IFA with intact/not intact RC: 12/32	IFA with intact RC: 46
Hao 20230^a^ [8]	ATSA	POA with Intact RC and preoperative ER > 0	
	ATSA	POA with Intact RC and preoperative ER ≤ 0	
Haritinian 2020 [28]	ATSA	POA/AVN with intact RC: 10/2	POA/AVN with intact RC: 38/1
Kany 20210^b^ [30]	ATSA	POA/IA/other: 3/2/6	POA/IA/other: 45/32/0
Kiet 2015 [31]	ATSA	RCTA	POA
Kirsch 2022 [32]	ATSA	POA with intact RC (Walch classification A/B/C/D): 24/38/3/2	POA with intact RC (Walch classification A/B/C/D): 26/37/3/1
Kleim 20210^b^ [33]	ATSA	POA/RCTA/IRCT: 6/25/1	POA: 23
Lawrence 2019 [35]	ATSA	RCTA	POA
Loew 2023 [37]	ATSA	RCTA/POA/PTA/RA: 74/12/29/2	POA/PTA/RA/AVN: 122/30/7/3
Luthringer 2022 [38]	ATSA	PHF and post‐traumatic fracture sequelae (had/not had undergone ORIF): 30/53	PHF and post‐traumatic fracture sequelae (had/not had undergone ORIF): 3/14
Magosch 2017 [9]	ATSA	Walch B2‐type glenoid arthropathy	
McLaughlin 20220^a^ [39]	ATSA	AVN	
	ATSA	RCTA	POA
Merolla 2020 [40]	ATSA	POA with intact RC	
Mowbary 2022 [41]	ATSA	RCTA/POA/PHF/AVN/RA/Dislocation/IFA: 2171/1599/480/349/238/53/37	NR
Nazzal, E. M.2023^a^ [10]	ATSA	POA with intact RC	POA with intact RC
	ATSA	POA with not intact RC	
Parada 2021 [42]	ATSA	Exclude PHF	
Polisetty 2021 [43]	ATSA	POA with intact RC	
Polisetty 2023 [44]	ATSA	POA with intact RC (Walch B2/B3 glenoids): 70/31	POA with intact RC (Walch B2/B3 glenoids): 70/31
Poondla 2020^a^ [45]	ATSA	RCTA with age between 50 and 69 years	POA with age between 50 and 69 years
	ATSA	RCTA with age between 70 and 89 years	POA with age between 70 and 89 years
Postacchini 2015 [46]	ATSA	IRCT/severe RCTA: 5/7	POA: 12
Schaller 2022 [48]	ATSA	RCTA	Intact RC
Schoch 2022 [49]	ATSA	NR	
Simovitch 2017 [51]	ATSA	POA with RCTA or RC tear	POA
Triplet 2015 [53]	ATSA	RCTA	POA

Abbreviations: AVN, avascular necrosis; ER, external rotation; IA, instability arthropathy; IFA, inflammatory arthropathy; IRCT, irreparable rotator cuff tear; PHF, proximal humeral fracture; POA, primary osteoarthritis; PTA, post‐traumatic arthritis; RA, rheumatoid arthritis; RC, rotator cuff; RCTA, rotator cuff tear arthropathy.

^a^ There were different subgroups in the study, among which Cox 2019 was grouped according to dominant hand or not, and the other grouping methods were shown in the baseline data section of the table.

^b^ The study included data on RTSA compared with HA and ATSA.

### 3.3 Comparison of Postoperative Function Not Included in Meta‐Analysis

In the study by Kany et al. [30], the Constant score for the HA carbon head subgroup was better than that of the RTSA group postoperatively, while the remaining RTSA group demonstrated superior functional scores compared to the HA group. In terms of postoperative mobility, the RTSA group exhibited significantly better forward flexion and abduction compared to the HA group, whereas internal and external rotation were inferior to those in the HA group. However, it should be noted that the study by Batar et al. [16] suggested that the external rotation function was superior in the RTSA group, but this may be attributed to the different measurement method (at 90° abduction) compared to other studies as shown in Table SII. When comparing the RTSA group to the ATSA group, the findings regarding postoperative functional scores and changes in functional scores were mixed between the two groups. In terms of postoperative forward flexion, abduction and external rotation function, only Luthringer et al. [38] suggested that the RTSA group was superior to the ATSA group in postoperative forward flexion function, while the rest showed that the ATSA group was superior. The amount of change before and after surgery was also mixed. There was no significant difference in internal rotation function between the two groups, but the ATSA group showed more significant changes before and after surgery as shown in Table SIII.

### 3.4 Postoperative Outcome Index

The results of 10 studies [7, 10, 21, 23, 26, 31, 32, 39, 44, 45] revealed no statistically significant difference in VAS score between RTSA group (*n* = 808) and ATSA group (*n* = 1189) (*I* ^2^ = 68.9%; 95% CI: −0.35 to 0.26 point; *p* = 0.78). However, the Constant score in the ATSA group (*n* = 5848) was significantly higher than that in the RTSA group (*n* = 4224) based on the findings of 12 studies [8, 9, 23, 24, 25, 28, 38, 39, 45, 46, 49, 51] (*I* ^2^ = 85.5%; 95% CI: 4.79, 0.93; *p* = 0.004). Similarly, the ASES score in the ATSA group (*n* = 6226) was significantly higher than that in the RTSA group (*n* = 4572) according to the results of 16 studies [7, 8, 10, 21, 23, 24, 25, 26, 31, 32, 38, 39, 44, 45, 49, 51] (*I* ^2^ = 77.3%; 95% CI: 4.94, 1.38; *p* = 0.001).

Regarding the UCLA scores, seven studies [8, 24, 25, 38, 39, 49, 51] found no statistically significant difference between the RTSA group (*n* = 3764) and the ATSA group (*n* = 5022) (*I* ^2^ = 56.2%; 95% CI: −0.34 to 0.57 point; *p* = 0.616). However, based on the findings of nine studies [8, 21, 24, 25, 26, 38, 39, 49, 51], the SST score of ATSA group (*n* = 5136) was significantly better than that in RTSA group (*n* = 3893) (*I* ^2^ = 27.7%; 95% CI: −0.61 to −0.28 point; *p* < 0.0001).

The results of 17 studies [7, 8, 9, 10, 23, 24, 25, 26, 28, 31, 32, 38, 39, 43, 44, 46, 51] showed that the ATSA group (*n* = 3408) had significantly better postoperative forward flexion function compared to the RTSA group (*n* = 2607) (*I* ^2^ = 48.4%; 95% CI: −6.14 to −2.25; *p* < 0.0001). Additionally, the active abduction angle in the ATSA group (*n* = 2552) was significantly better than that in the RTSA group (*n* = 2060) based on the results of 10 studies [8, 9, 24, 25, 28, 31, 38, 39, 46, 51] (*I* ^2^ = 80.2%; 95% CI: −10.75 to −1.41; *p* = 0.011).

After screening, 10 studies [8, 10, 24, 25, 26, 28, 38, 39, 43, 44] with similar recording methods were included in the meta‐analysis. These studies utilized the vertebral segment score counting method for hand internal rotation touch (hip or greater trochanter: 2 points; Sacrum to L4: 4; L3‐L1: 6 points; T12‐T8: 8 points; T7‐T1), and the statistical analysis showed that the postoperative internal rotation angle in the ATSA group (*n* = 2484) was significantly better than that in the RTSA group (*n* = 1584) (*I* ^2^ = 58.5%; 95% CI: −0.79 to −0.58; *p* < 0.0001). Moreover, the results of 17 studies [7, 8, 9, 10, 23, 24, 25, 26, 28, 31, 32, 38, 39, 43, 44, 46, 51] indicated that the postoperative external rotation angle in ATSA group (*n* = 34.08) was significantly better than that in RTSA group (*n* = 2562) with (*I* ^2^ = 75.4%; 95% CI: −11.65 to 7.43; *p* < 0.0001) as shown in Table 3.

**TABLE 3:** Meta‐regression analysis of outcomes with large heterogeneity in RTSA group versus ATSA group was summarized.

Covariates	No. of study	No. of group	Coefficient	Standard error	*t* score	*p*	95% CI
VAS score							
Disease	9	12	0.849	0.376	2.26	0.065	−0.071 to 1.770
Region	9	12	0.030	0.267	0.11	0.913	−0.624 to 0.684
Age	8	11	−0.567	0.375	−1.51	0.181	−1.484 to 0.351
Sex	8	11	−0.980	0.729	−1.34	0.227	−2.764 to 0.804
Follow‐time	9	12	−0.075	0.376	−0.20	0.847	−0.996 to 0.845
Constant score							
Disease	12	15	−2.593	2.542	−1.02	0.334	−8.344 to 3.157
Region	12	15	0.857	0.766	1.12	0.292	−0.876 to 2.589
Age	12	15	8.783	2.898	3.03	0.014^a^	2.228 to 15.339
Sex	12	15	4.027	4.651	0.87	0.409	−6.494 to 14.549
Follow‐time	12	15	−1.266	2.687	−0.47	0.649	−7.344 to 4.812
ASES score							
Disease	15	20	−6.807	2.231	−3.05	0.009^a^	−11.593 to 2.022
Region	15	20	1.096	1.276	0.86	0.405	−1.640 to 3.833
Age	14	19	4.827	2.209	2.18	0.046^a^	0.088 to 9.565
Sex	14	19	8.483	4.351	1.95	0.072	−0.849 to 17.815
Follow‐time	14	19	2.628	2.141	1.23	0.240	−1.964 to 7.219
UCLA score							
Disease	7	8	−0.916	0.431	−2.13	0.124	−2.287 to 0.456
Region	7	8	0.232	0.208	1.12	0.345	−0.429 to 0.894
Sex	7	8	−0.122	0.663	−0.18	0.865	−2.231 to 1.987
Follow‐time	7	8	−1.979	1.228	−1.61	0.205	−5.886 to 1.929
Forward flexion							
Disease	17	21	−4.846	3.186	−1.52	0.151	−11.679 to 1.988
Region	17	21	0.108	1.021	0.11	0.918	−2.083 to 2.298
Age	16	20	4.999	3.412	1.47	0.615	−2.318 to 12.318
Sex	16	20	6.865	7.048	0.97	0.347	−8.251 to 21.982
Follow‐time	17	21	−11.248	8.366	−1.34	0.200	−6.539 to 6.226
Abduction							
Disease	9	11	−4.493	3.468	−1.30	0.252	−13.409 to 4.422
Region	9	11	−1.991	1.470	−1.36	0.233	−5.769 to 1.786
Age	8	10	−1.477	7.993	−0.18	0.861	−22.023 to 19.068
Sex	8	10	−9.838	11.977	−0.82	0.449	−40.626 to 20.950
Follow‐time	9	11	−19.710	6.455	−3.05	0.028^a^	−36.304 to −3.116
Internal rotation							
Disease	10	13	−0.206	0.146	−1.40	0.203	−0.552 to 0.141
Region	10	13	0.164	0.069	2.38	0.049^a^	0.001 to 0.328
Age	10	13	0.707	0.253	2.79	0.027^a^	0.108 to 1.306
Sex	10	13	0.229	0.442	0.52	0.621	−0.817 to 1.275
Follow‐time	10	13	0.534	0.171	3.12	0.017^a^	0.129 to 0.939
External rotation							
Disease	17	21	−5.759	2.623	−2.19	0.046^a^	−11.387 to −0.132
Region	17	21	−0.815	0.758	−1.07	0.301	−2.441 to 0.811
Age	16	20	2.698	2.863	0.94	0.362	−3.443 to 8.838
Sex	16	20	6.082	6.047	1.01	0.332	−6.888 to 19.052
Follow‐time	17	21	−3.161	2.448	−1.29	0.217	−8.410 to 2.087

^a^ There is a significant difference and considering this index is the cause of the large heterogeneity between the studies.

Through subgroup analysis based on the proportion of rotator cuff injuries, we observed the following findings: (1) In the subgroup of the “same disease” with proportion of rotator cuff injuries between the two groups were no statistically significant differences, there were no statistically significant differences in postoperative Constant scores (*I* ^2^ = 0%; 95% CI: −1.86 to 1.32 points; *p* = 0.74), ASES scores (*I* ^2^ = 53.4%; 95% CI: −2.02 to 3.89 points; *p* = 0.543), and range of outreach activities. However, the VAS score of the RTSA group was better than that of the ATSA group (*I* ^2^ = 0%; 95% CI: −0.96 to −0.17 points; *p* = 0.005). Other indicators in the RTSA group were worse than the ATSA group, including SST scores (*I* ^2^ = 27.7%; 95% CI: −0.61 to −0.28 points; *p* < 0.0001), forward bending function (*I* ^2^ = 17%; 95% CI: −5.24 to −0.78 points; *p* = 0.008), internal rotation function (*I* ^2^ = 50.3%; 95% CI: −1.13 to 0.54 points; *p* < 0.0001), and outward rotation function (*I* ^2^ = 77.4%; 95% CI: −11.05 to −4.22 points; *p* < 0.0001). (2) In the subgroup of “different diseases” with proportion of rotator cuff injuries between the two groups were statistically significant differences, except for postoperative UCLA scores and VAS scores, there were significant differences in other indicators, with the RTSA group showing significantly worse outcomes compared to the ATSA group. The magnitude of difference between the two groups was greater than that observed in the subgroup with the same disease. Specifically, there were significant differences in Constant scores (*I* ^2^ = 89.8%; 95% CI: −6.26 to −1.28 points; *p* < 0.0001), ASES scores (*I* ^2^ = 79.1%; 95% CI: −6.87 to −2.70 points; *p* < 0.0001), SST scores (*I* ^2^ = 40.5%; 95% CI: −0.70 to −0.28 points; *p* < 0.0001), forward flexion function (*I* ^2^ = 60.3%; 95% CI: −8.53 to −2.44 points; *p* < 0.0001), abduction function (*I* ^2^ = 82.5%; 95% CI: −13.35 to −1.35 points; *p* = 0.016), internal rotation function (*I* ^2^ = 68.3%; 95% CI: −1.11 to −0.41 points; *p* < 0.0001), and external rotation function (*I* ^2^ = 64.7%; 95% CI: −13.52 to −8.68 points; *p* < 0.0001) as shown in Table 4 and Figures [Link], [Link].

**TABLE 4:** Results of meta‐analysis of postoperative outcome indicators in RTSA group versus ATSA group.

Covariates	No. of study	No. of group	No. of shoulders RTSA/ATSA	Point estimate	Heterogeneity (*I* ^2^ value, %)	95% confidence intervals	
						Range	*p* (favors group)
VAS score							
Same disease	3	3	192/246	−0.56	0.00	−0.96 to −0.17	0.005 (RTSA)
Different disease	8	10	616/943	0.10	71.6	−0.26 to 0.47	0.573 (no sig.)
Overall	10	13	808/1189	−0.04	68.9	−0.35 to 0.26	0.780 (no sig.)
Constant score							
Same disease	4	5	457/1205	−0.27	0.00	−1.86 to 1.32	0.740 (no sig.)
Different disease	9	10	3767/4463	−3.78	89.8	−6.26 to −1.28	0.003 (ATSA)
Overall	12	15	4224/5848	−2.86	85.5	−4.79 to −0.93	0.004 (ATSA)
ASES score							
Same disease	5	6	607/1341	0.94	53.4	−2.02 to 3.89	0.543 (no sig.)
Different disease	13	15	3965/4885	−4.78	79.1	−6.87 to −2.70	< 0.0001 (ATSA)
Overall	16	21	4572/6226	−3.16	77.3	−4.94 to −1.38	0.001 (ATSA)
UCLA score							
Same disease	2	3	415/1080	0.91	62.0	−0.29 to 2.10	0.137 (no sig.)
Different disease	5	5	3349/3942	−0.16	17.7	−0.48 to 0.16	0.326 (no sig.)
Overall	7	8	3764/5022	0.12	56.2	−0.34 to 0.57	0.616 (no sig.)
SST score							
Same disease	2	3	415/1080	−0.31	0.00	−0.61 to −0.01	0.042 (ATSA)
Different disease	8	8	3478/4056	−0.49	40.5	−0.70 to −0.28	< 0.0001 (ATSA)
Overall	10	11	3893/5136	0.44	27.7	−0.61 to −0.28	< 0.0001 (ATSA)
FE							
Same disease	8	9	715/1718	−3.01	17.0	−5.24 to −0.78	0.008 (ATSA)
Different disease	11	12	1892/1690	−5.48	60.3	−8.53 to −2.44	< 0.0001 (ATSA)
Overall	17	21	2607/3408	−4.20	48.4	−6.14 to −2.25	< 0.0001 (ATSA)
AB							
Same disease	4	5	460/1205	−4.04	61.9	−10.74 to 2.67	0.238 (no sig.)
Different disease	7	7	1600/1347	−7.35	82.4	−13.35 to −1.35	0.016 (ATSA)
Overall	10	12	2060/2552	−6.08	80.2	−10.75 to −1.41	0.011 (ATSA)
IR							
Same disease	6	7	615/1565	−0.84	50.3	−1.13 to −0.54	< 0.0001 (ATSA)
Different disease	6	6	969/919	−0.76	68.3	−1.11 to −0.41	< 0.0001 (ATSA)
Overall	10	13	1584/2484	−0.79	58.5	−0.79 to −0.58	< 0.0001 (ATSA)
ER							
Same disease	8	9	715/1718	−7.63	77.4	−11.05 to −4.22	< 0.0001 (ATSA)
Different disease	11	12	1847/1690	−11.10	64.7	−13.52 to −8.68	< 0.0001 (ATSA)
Overall	17	21	2562/3408	−9.54	75.4	−11.65 to −7.43	< 0.0001 (ATSA)

*Note*: No sig.: There was no significant difference between the two groups.

The findings from three studies [17, 20, 27] indicated that the ASES score in RTSA group (*n* = 98) was significantly higher than that in HA group (*I* ^2^ = 29.6%; 95% CI: 3.73 to 19.02 point; *p* = 0.004). However, the results of the three studies [2, 20, 27] showed that the postoperative SST score in the RTSA group (*n* = 100) was higher than that in the HA group (*n* = 95), but the difference was not statistically significant (*I* ^2^ = 0; 95% CI: −0.15 to 1.62 point; *p* = 0.102). In terms of functional outcomes, the results of six studies [17, 19, 20, 29, 47, 54] showed that the postoperative flexion function in the RTSA group (*n* = 249) was significantly better than that in the HA group (*n* = 269), with a statistically significant difference (*I* ^2^ = 49%; 95% CI: 14.72 to 34.57; *p* < 0.0001). Additionally, the active abduction angle in the RTSA group (*n* = 93) was significantly better than that in the HA group (*n* = 95) based on the results of three studies [17, 29, 47] (*I* ^2^ = 51.9%; 95% CI: 16.24 to 43.93; *p* < 0.0001). However, the results of the four studies [17, 19, 29, 47] showed that the postoperative external rotation angle in the HA group (*n* = 152) was better than that in the RTSA group (*n* = 134), but the difference was not statistically significant (*I* ^2^ = 56.8%; 95% CI: −10.31 to 1.25; *p* = 0.124) as shown in Figures [Link], [Link].

### 3.5 Complications and Their Incidences

The main complications observed in this study include prosthesis loosening and instability, infection, nerve damage, and intraoperative or postoperative fractures. It was found that patients with nerve injuries can recover spontaneously during postoperative rehabilitation, as indicated in Tables 3 and SI. By analyzing four serious postoperative complications, it was observed that the incidence rate was higher in the HA group compared to the other two groups. The RTSA group had a lower incidence of postoperative prosthesis loosening and instability compared to the ATSA group. However, the incidence of postoperative or intraoperative fractures was significantly higher in the RTSA group than in the ATSA group. The incidence of infection was similar between the two groups. Additionally, the ATSA group had a higher incidence of rotator cuff injury (2.87%), as shown in Table 5.

**TABLE 5:** Summary of four kinds of serious complications after operation.

Complications	No. of RTSA group (no. of study)	No. of HA group (no. of study)	No. of ATSA group (no. of study)
Prosthetic loosening OR instability	123/4949 (13)	7/96 (2)	114/2879 (11)
Infection	61/4886 (14)	10/241 (7)	38/3054 (8)
Fracture	149/5418 (21)	3/93 (2)	19/3047 (9)
Rotator cuff injury	3/64 (1)	12/120 (3)	97/3375 (14)

**FIGURE 2:**
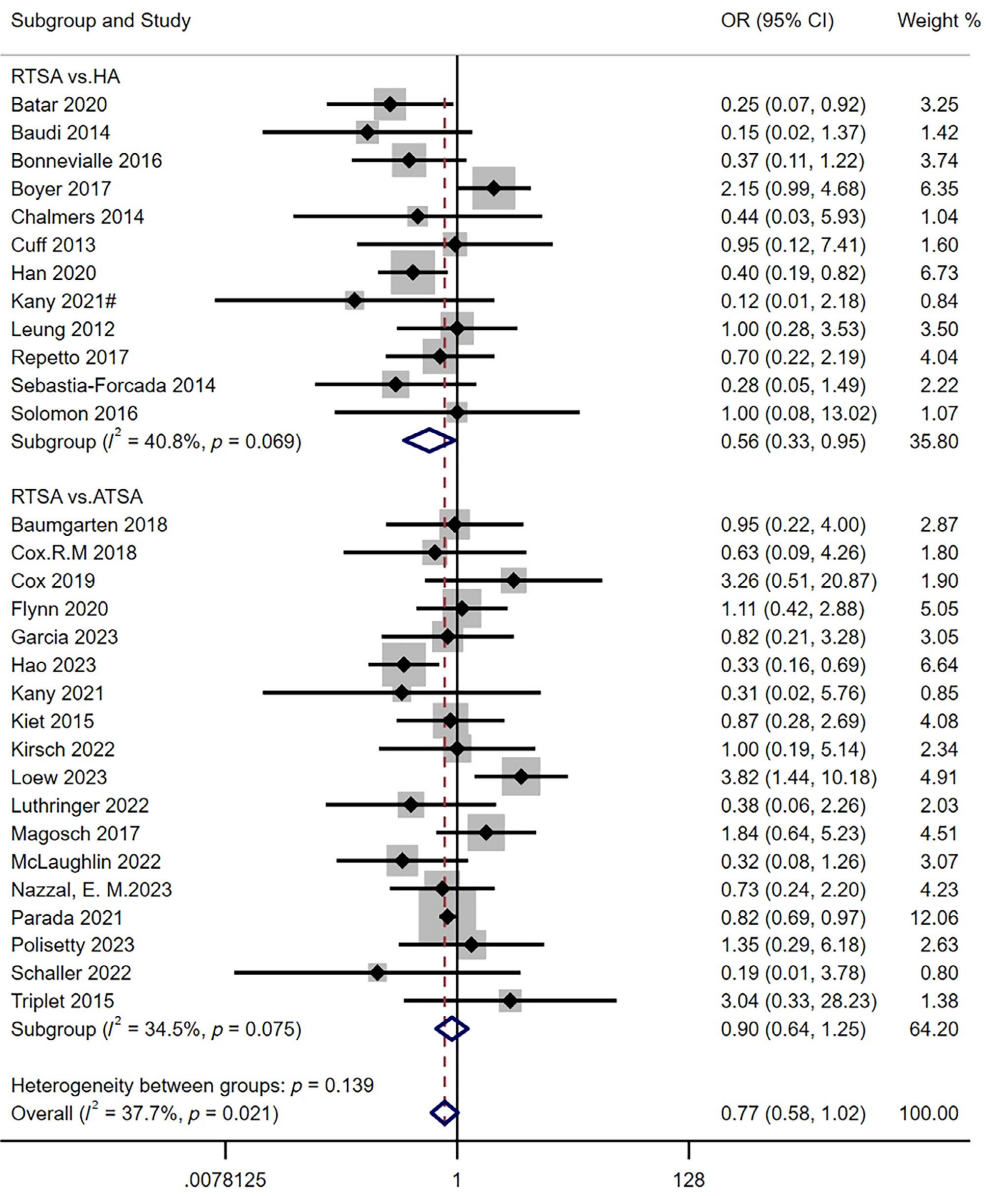
Comparison of rate of complications between RTSA group and other two group. OR, odds ratio; CI, confidence interval.

The results of 12 studies [4, 16, 17, 19, 20, 22, 27, 30, 36, 47, 50, 52] showed that the incidence of postoperative complications in RTSA group (*n* = 366) was significantly lower than that in HA group (*n* = 405), and the difference was statistically significant (*I* ^2^ = 40.8%; 95% CI: 0.56 to 0.95; *p* = 0.032). The results of 18 studies [7, 8, 9, 10, 18, 21, 25, 26, 30, 31, 32, 37, 38, 39, 42, 44, 48, 53] showed that the incidence of postoperative complications in RTSA group (*n* = 5532) was lower than that in ATSA group (*n* = 4456), but the difference was not statistically significant (*I* ^2^ = 34.5%; 95% CI: 0.64 to 1.25; *p* = 0.521). Combined statistics showed that the incidence of postoperative complications in RTSA group was lower than that in control group, but the difference was not statistically significant (*I* ^2^ = 37.7%; 95% CI: 0.58 to 1.02; *p* = 0.065) as shown in Figure 2.

### 3.6 The Incidence of Reoperation

**FIGURE 3:**
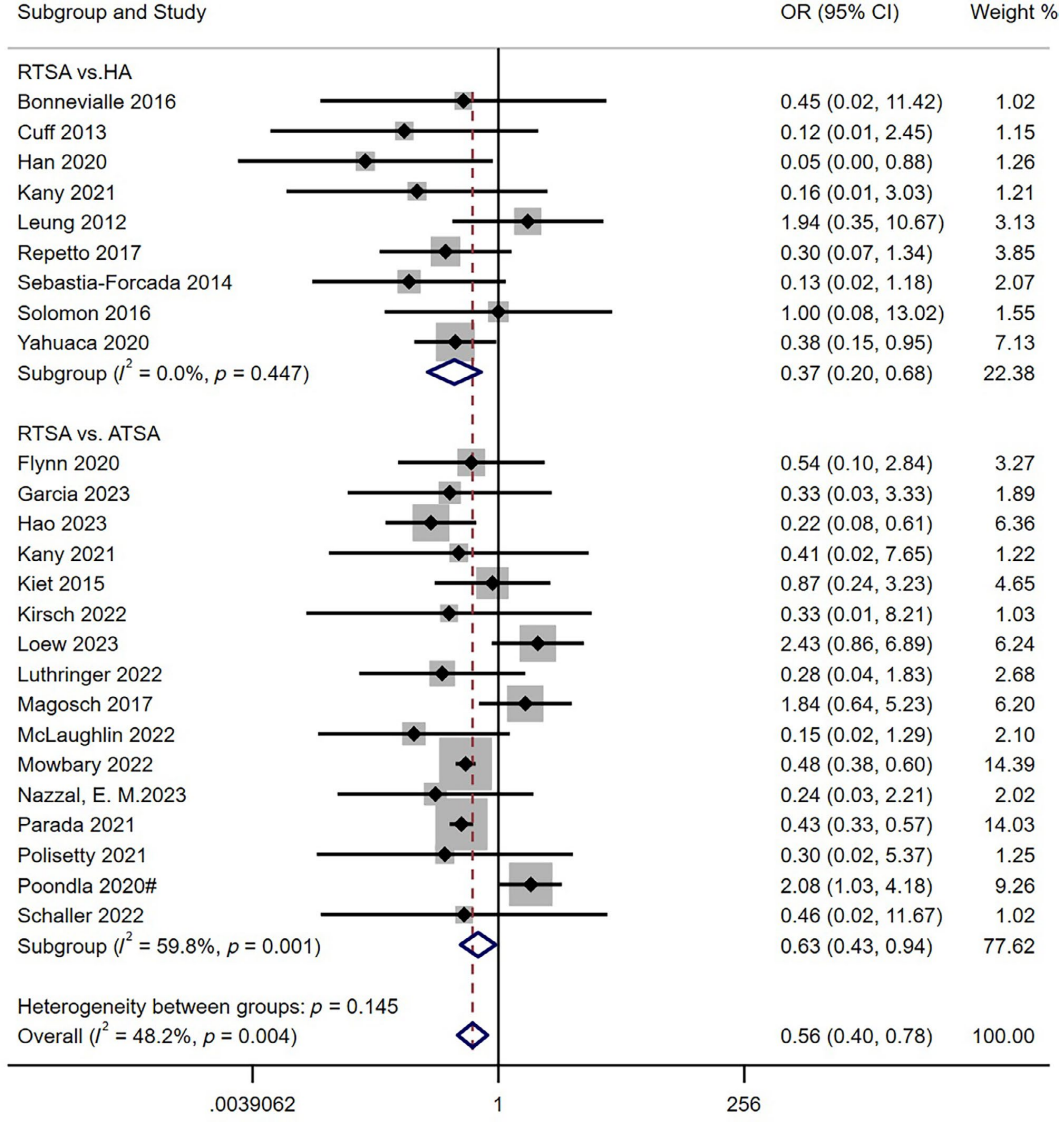
Comparison of rate of reoperation between RTSA group and other two group. OR, odds ratio; CI, confidence interval.

The results of nine studies [19, 22, 27, 30, 36, 47, 50, 52, 54] showed that the rate of reoperation in RTSA group (*n* = 340) was significantly lower than that in HA group (*n* = 382), with a statistically significant difference (*I* ^2^ = 0%; 95% CI: 0.20 to 0.68; *p* = 0.001). Two large‐scale data studies also analyzed the comparative results of postoperative reoperation rates between RTSA and HA. However, due to various limitations such as high mortality rate, data loss [56], and statistical differences among time periods and other studies, these data could not be included in the meta‐quantitative analysis [57]. Nonetheless, these studies showed that the rate of reoperation in the RTSA group was lower than that in the HA group, with a per 100 component‐years ratio of 0.58 (RTSA) versus 1.16 (HA) [56] and reoperation rates of 7.0% (RTSA) compared to 11.7% (HA) [57]. Furthermore, the results from 16 studies [8, 9, 10, 25, 26, 30, 31, 32, 37, 38, 39, 41, 42, 43, 45, 48] showed that the incidence of reoperation after surgery in RTSA group (*n* = 10,246) was lower than that in ATSA group (*n* = 8419), and the difference between the two groups was statistically significant (*I* ^2^ = 59.8%; 95% CI: 0.63 to 0.94; *p* = 0.024). The combined effect size showed that the rate of reoperation in the RTSA group was lower than that in the control group, and the difference was statistically significant (*I* ^2^ = 48.2%; 95% CI: 0.40 to 0.78; *p* = 0.001) as shown in Figure 3.

### 3.7 Heterogeneity and Publication Bias Analysis

Due to limited available data, the meta‐analysis did not assess heterogeneity and publication bias in the comparison of postoperative functional scores and activity between RTSA and HA. Subgroup analysis revealed that differences in treated diseases contributed to the significant heterogeneity in postoperative functional scores between RTSA and ATSA studies, as shown in Table 4. Regression analysis on factors such as disease, age, gender, follow‐up time, and study location showed that age differences were responsible for the significant heterogeneity in Constant score, ASES score, and internal rotation function after surgery. Subgroup analysis based on age differences indicated that ATSA was superior to RTSA in all subgroups, with statistical significance (*p* < 0.05). Subgroup analysis of internal and external rotation based on follow‐up time differences showed that, at the same follow‐up time, there was no statistical significance in abduction function between the two groups. However, in other subgroups, ATSA demonstrated better outcomes compared to the RTSA group, with statistical significance (*p* < 0.05). Geographical location appeared to contribute to the significant heterogeneity in internal rotation function, but the results consistently favored ATSA over RTSA without requiring further subgroup analysis, as shown in Table 3. The Egger test indicated no publication bias among the outcome indicators in each study, as shown in Table 6.

**TABLE 6:** Egger detection of the outcome indicators with a large number of included studies shows that there is no publication bias in each outcome indicator.

Covariates	No. of study	No. of group	*t* score	*p*
VAS score	10	13	0.38	0.713
Constant score	12	15	−0.62	0.545
ASES score	15	20	−1.14	0.269
UCLA score	7	8	1.84	0.115
SST score	9	11	0.05	0.965
Forward flexion	17	21	−0.82	0.421
Abduction	10	12	1.90	0.087
Internal rotation	10	13	−2.18	0.052
External rotation	17	21	1.04	0.312
Complication rate^a^	12	12	−1.27	0.234
Complication rate	18	18	0.40	0.694
Reoperation rate^a^	9	9	−0.77	0.465
Reoperation rate	16	16	0.55	0.590

^a^ RTSA versus HA.

## 4 Discussion

### 4.1 Summary of Main Results

This meta‐analysis indicated that RTSA yielded better outcomes than HA across various measures, except for external rotation function, aligning with findings from Shukla et al. [13]. When comparing RTSA to ATSA, RTSA demonstrated advantages in postoperative UCLA scores, complication rates, and revision rates, while other measures favored the ATSA group. However, subgroup analysis indicated that when disease treatments were comparable, differences in postoperative functional scores and range of motion between RTSA and ATSA were minimal or not statistically significant, likely due to lower baseline scores in shoulder arthritis patients with concurrent rotator cuff injuries. Overall, despite significant differences in treated conditions, ATSA remained superior to RTSA. Regression and subgroup analyses assessed the effects of study region, age, gender distribution, and follow‐up duration on outcome heterogeneity, and even after adjusting for these factors, ATSA continued to demonstrate overall superiority. Nonetheless, RTSA showed a lower postoperative complication rate without a statistically significant difference (*p* = 0.521) and a statistically significant difference in revision rates (*p* = 0.024).

### 4.2 Advantages and Applications of RTSA

In clinical practice, complex proximal humerus fractures, classified as Neer 3–4 part fractures, present significant challenges due to severe morphological deformation, compromised blood supply to the humeral head, and difficulties in achieving reduction. Traditional reconstruction surgery with locking plates carries a higher risk of complications, including avascular necrosis of the humeral head, nonunion, and screw failure, frequently requiring revision surgery [58]. The introduction of shoulder arthroplasty has offered an effective treatment option for these patients. Initially, HA was the primary choice; however, subsequent research has indicated that optimal functional recovery is significantly dependent on the healing of the greater tuberosity, which is crucial for the biomechanics of the rotator cuff [59, 60]. The emergence of RTSA has introduced a new replacement option that diminishes reliance on the rotator cuff for functional recovery. Over the past decade, the utilization rate of RTSA has exceeded that of HA, establishing it as the primary treatment for complex proximal humerus fractures [57, 61]. In the management of end‐stage shoulder arthritis, ATSA was historically common; however, functional recovery following ATSA is heavily reliant on the rotator cuff. Given that end‐stage arthritis often involves varying degrees of rotator cuff damage, this creates a significant risk of compromised rotator cuff function and poor prognosis [28]. Young et al. [62] demonstrated a marked increase in the incidence of secondary rotator cuff tears post‐ATSA over time. Additionally, Shields, Ho, and Wiater [63] reported a high prevalence of subscapularis muscle dysfunction after ATSA, which can result in postoperative prosthesis instability and pain. These issues contribute to the elevated revision rates associated with ATSA, aligning with our study's findings. Consequently, RTSA has gained widespread acceptance as the preferred treatment for end‐stage shoulder arthritis.

### 4.3 Complications and Limitations of RTSA

Hanisch et al. [64] reported that patients under 65 undergoing RTSA do not face an increased risk of complications, revisions, or adverse outcomes compared to older patients. Kany et al. [30] found no significant difference in postoperative function between RTSA and ATSA in patients under 50 years old. Furthermore, RTSA demonstrated significantly lower rates of complications and revisions compared to HA, although a metal head was associated with poorer postoperative function than a carbon head. Conversely, Aibinder et al. [65] suggested that younger age is a risk factor for higher complications and revision rates in RTSA, likely due to greater activity demands. Chelli et al. [66] found that among patients with primary osteoarthritis (OA) and rotator cuff injuries, younger patients experienced higher complication and revision rates. Specifically, 75.7% of patients aged 60 or younger underwent RTSA without complications or revisions within 10 years, compared to 88.8%, 91.3%, and 94.3% for those aged 60–69, 70–79, and over 80, respectively. Forlizzi et al. [67] proposed that the preoperative diagnosis of primary OA is the strongest predictor of excellent clinical outcomes after RTSA. Christophe et al. [68] reported a 96% survival rate without revision for RTSA in rheumatoid arthritis patients over a 7‐year follow‐up. Pettit et al. [69] investigated the influence of different shoulder morphologies on postoperative function after RTSA, concluding that good outcomes could be achieved regardless of deformity morphology over a 2‐year follow‐up.

While RTSA presents theoretical design advantages and favorable clinical outcomes, its superiority over traditional HA and ATSA requires further exploration in clinical practice. RTSA involves significant destruction of the shoulder joint structure, complicating remedial efforts in case of failure. This is reflected in the notable postoperative complications associated with RTSA, including a 2.75% risk of intraoperative or postoperative fractures. Although its complication and revision rates are lower than those of other procedures, they are still considerable. For patients with failed HA or ATSA, RTSA can serve as a revision option, as it inflicts less damage to the glenoid [70, 71], thereby promoting improved post‐revision function.

Several meta‐analyses [11, 12, 13, 72, 73, 74, 75, 76] have indicated the superior efficacy of RTSA, demonstrating lower rates of complications and revision surgeries. However, these studies mainly addressed factors such as geriatric fractures and prosthesis placement and their influence on postoperative clinical outcomes. Rossi et al. [76] found no significant differences in clinical outcomes, complications, or revision rates between cemented and uncemented RTSA implants. Similarly, Larose et al. [73] reported comparable postoperative outcomes between Inlay and Onlay designs for humeral components, although Onlay implants exhibited a lower rate of scapular notching but a higher incidence of scapular fractures. Initial beliefs suggested that functional recovery in RTSA was independent of greater tuberosity healing; however, subsequent research indicates that healing of the greater tuberosity can enhance postoperative mobility [77, 78]. Therefore, the healing of the greater tuberosity should be a consideration in RTSA surgery, warranting further research to refine surgical techniques and approaches.

### 4.4 Comparison With Other Reviews

The aforementioned studies highlight the favorable clinical outcomes and expanding indications of RTSA from various perspectives. However, comparative efficacy data between RTSA and traditional HA and ATSA for specific indications remains insufficient. Our study aimed to provide a comprehensive summary and comparison by not strictly limiting baseline data, such as age, treated conditions, and follow‐up duration. This approach enabled us to leverage a larger pool of real‐world comparative data to assess the appropriateness of RTSA indications based on existing evidence. We conducted subgroup and meta‐regression analyses for variables with substantial study sizes and high heterogeneity to mitigate the influence of relevant baseline factors. Furthermore, Egger's test was employed to address publication bias. These measures support the evaluation of whether the indications for RTSA should be further expanded.

## 5 Conclusion

In summary, we advocate for the more frequent use of RTSA in elderly patients with 3‐ or 4‐part proximal humeral fractures. However, the evidence does not support RTSA as significantly superior to HA in younger patients. Given the increased trauma and challenges associated with revision surgeries, RTSA is not recommended for young patients with complex proximal humeral fractures without irreparable rotator cuff injuries. In cases of end‐stage shoulder arthritis and humeral head necrosis, RTSA exhibits good clinical efficacy with lower complication and revision rates. However, ATSA offers greater benefits in postoperative functional scores and range of motion. Considering the risks associated with revisions, we do not recommend expanding RTSA indications for complex shoulder joint diseases.

## 6 Limitations and Future Research

Our study has several limitations: (1) Due to the retrospective nature, only three randomized controlled trials were included, all with small sample sizes and inadequate data recording methods for meta‐analysis. (2) The inclusion of mixed baseline factors resulted in significant heterogeneity among studies. (3) Some studies had a long data collection period, with considerable elapsed time, as RTSA developed later than HA and ATSA, which may have led to less mature technology. Consequently, the results may not favor RTSA to some extent, although this does not reflect an inherent limitation of the procedure itself.

## Author Contributions

Huankun Li, Hangshen Bao, and Yonggen Zou conceived and designed the study. Huankun Li, Zhidong Yang, Yi Wang, and Yaocheng Pan performed the literature search, data extraction, and data analysis. Huankun Li, Jiayi Chen, Hongjun Chen, and Bisheng Shen helped with the data analysis and reviewed the manuscript for important content. Baijun Hu and Yonggen Zou supervised the study. All authors have read and approved the final version of the submitted version.

## Ethics Statement

The data utilized for this study were derived from databases that are publicly accessible and do not contain any personally identifiable information. Consequently, the typical ethical parameters pertaining to the use of human subjects in research do not apply.

## Conflicts of Interest

The authors declare no conflicts of interest.

## Supporting information


**Figure S1.** Comparison of postoperative ASES score between RTSA group and HA group. MD, mean difference; CI, confidence interval.

**Figure S2.** Comparison of postoperative SST score between RTSA group and HA group. MD, mean difference; CI, confidence interval.

**Figure S3.** Comparison of postoperative anterior flexion angle between RTSA group and HA group. MD, mean difference; CI, confidence interval.

**Figure S4.** Comparison of postoperative abduction angle between RTSA group and HA group. MD, mean difference; CI, confidence interval.

**Figure S5.** Comparison of postoperative external rotation angle between RTSA group and HA group. MD, mean difference; CI, confidence interval.

**Figure S6.** Comparison of postoperative VAS scores between RTSA group and ATSA group. MD, mean difference; CI, confidence interval.

**Figure S7.** Comparison of postoperative Constant scores between RTSA group and ATSA group. MD, mean difference; CI, confidence interval.

**Figure S8.** Comparison of postoperative ASES scores between RTSA group and ATSA group. MD, mean difference; CI, confidence interval.

**Figure S9.** Comparison of postoperative UCLA scores between RTSA group and ATSA group. MD, mean difference; CI, confidence interval.

**Figure S10.** Comparison of postoperative SST scores between RTSA group and ATSA group. MD, mean difference; CI, confidence interval.

**Figure S11.** Comparison of postoperative anterior flexion angle between RTSA group and ATSA group. MD, mean difference; CI, confidence interval.

**Figure S12.** Comparison of postoperative abduction angle between RTSA group and ATSA group. MD, mean difference; CI, confidence interval.

**Figure S13.** Comparison of postoperative internal rotation angle between RTSA group and ATSA group. MD, mean difference; CI, confidence interval.

**Figure S14.** Comparison of postoperative external rotation angle between RTSA group and ATSA group. MD, mean difference; CI, confidence interval.

**Table SI.** Postoperative complications and rehabilitation program.
**Table SII**. Summary of outcome measures not included in meta‐analysis (RTSA vs. HA).
**Table SIII**. Summary of outcome measures not included in meta‐analysis (RTSA vs. ATSA).

Data S1.

## Data Availability

The original contributions presented in the study are included in the article and Supporting Information. Further inquiries can be directed to the corresponding author.
