# Supplementary figures and images for: Whether the Indications for Reverse Shoulder Arthroplasty Should Continue to Be Expanded? A Systematic Review and Meta‐Analysis
Source: Orthop Surg. 2024 Dec 12;17(2):313–32. doi: 10.1111/os.14311 (PMC11787993; doi:10.1111/os.14311)

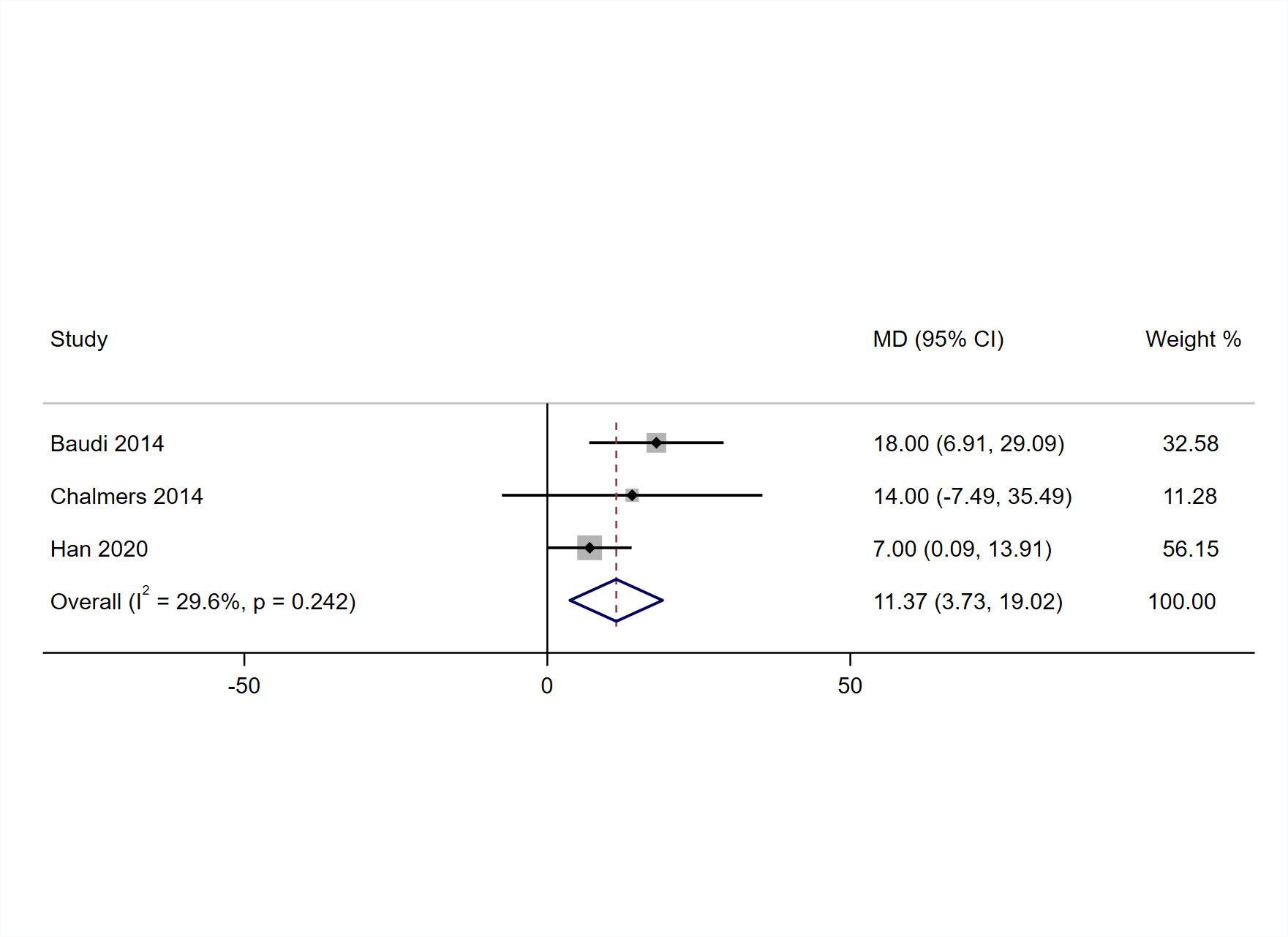

Supplement: Supplementary file 1 — Figure S1. Comparison of postoperative ASES score between RTSA group and HA group. MD, mean difference; CI, confidence interval. [file OS-17-313-s016.tif]

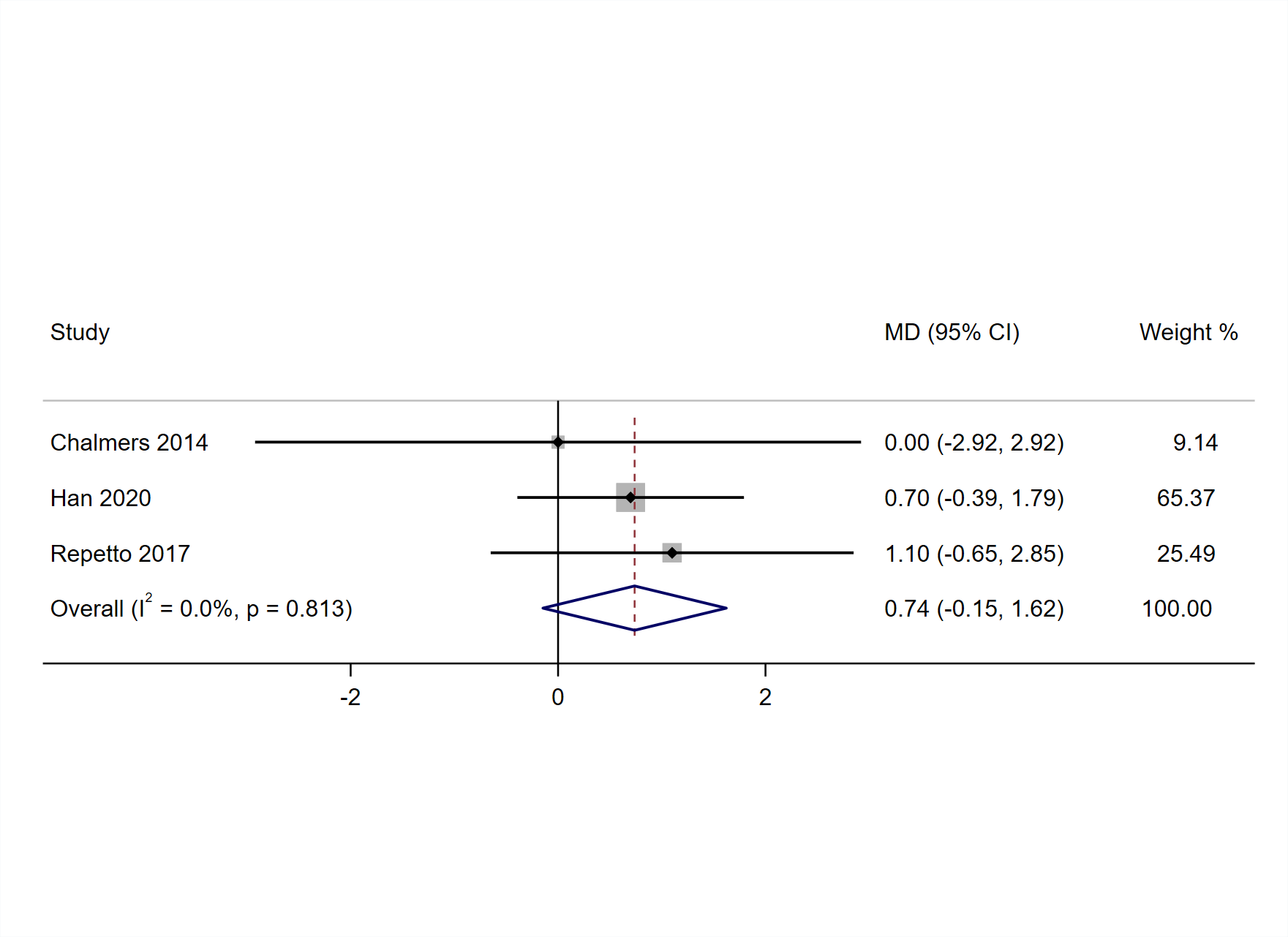

Supplement: Supplementary file 2 — Figure S2. Comparison of postoperative SST score between RTSA group and HA group. MD, mean difference; CI, confidence interval. [file OS-17-313-s003.tif]

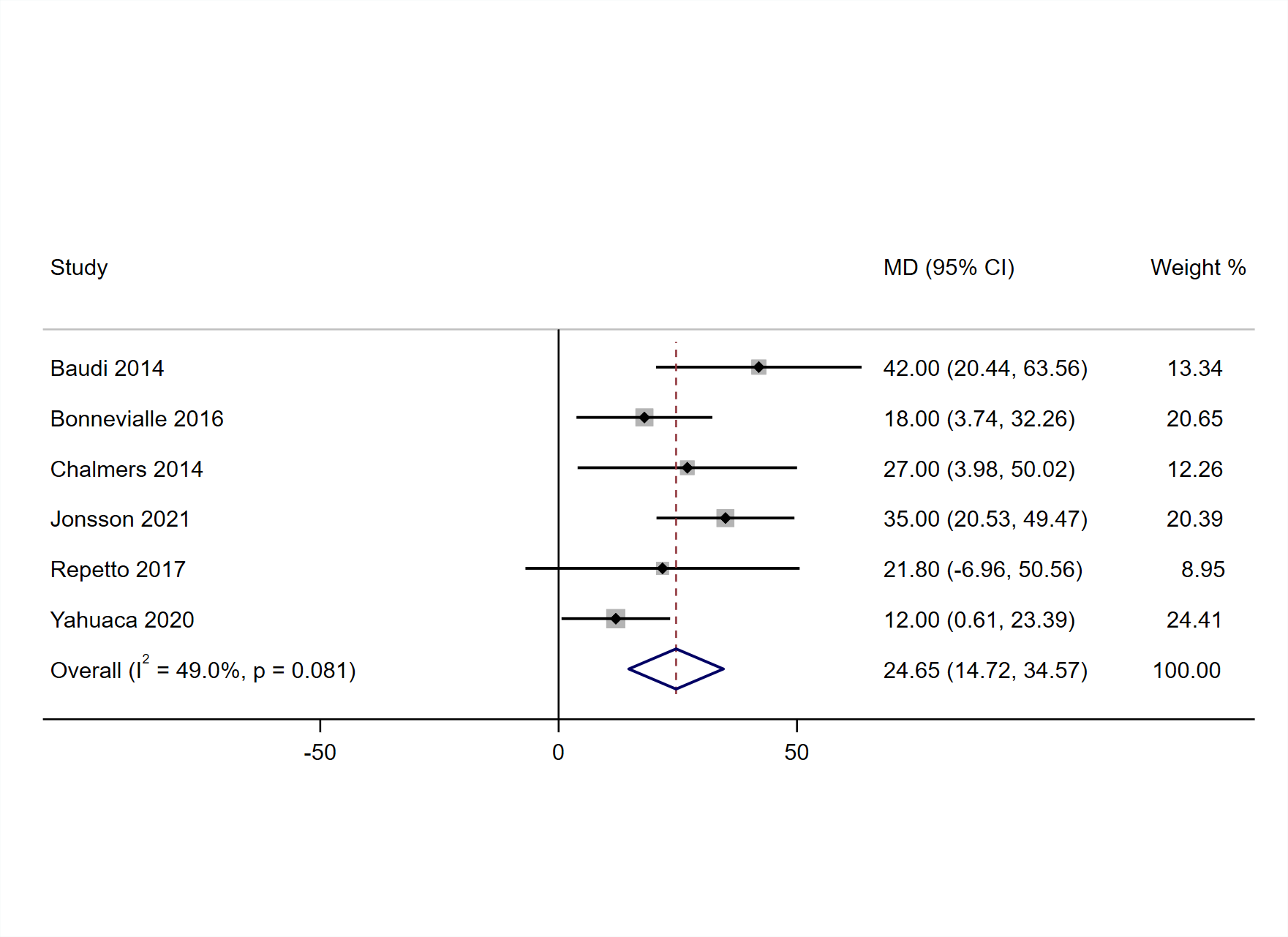

Supplement: Supplementary file 3 — Figure S3. Comparison of postoperative anterior flexion angle between RTSA group and HA group. MD, mean difference; CI, confidence interval. [file OS-17-313-s009.tif]

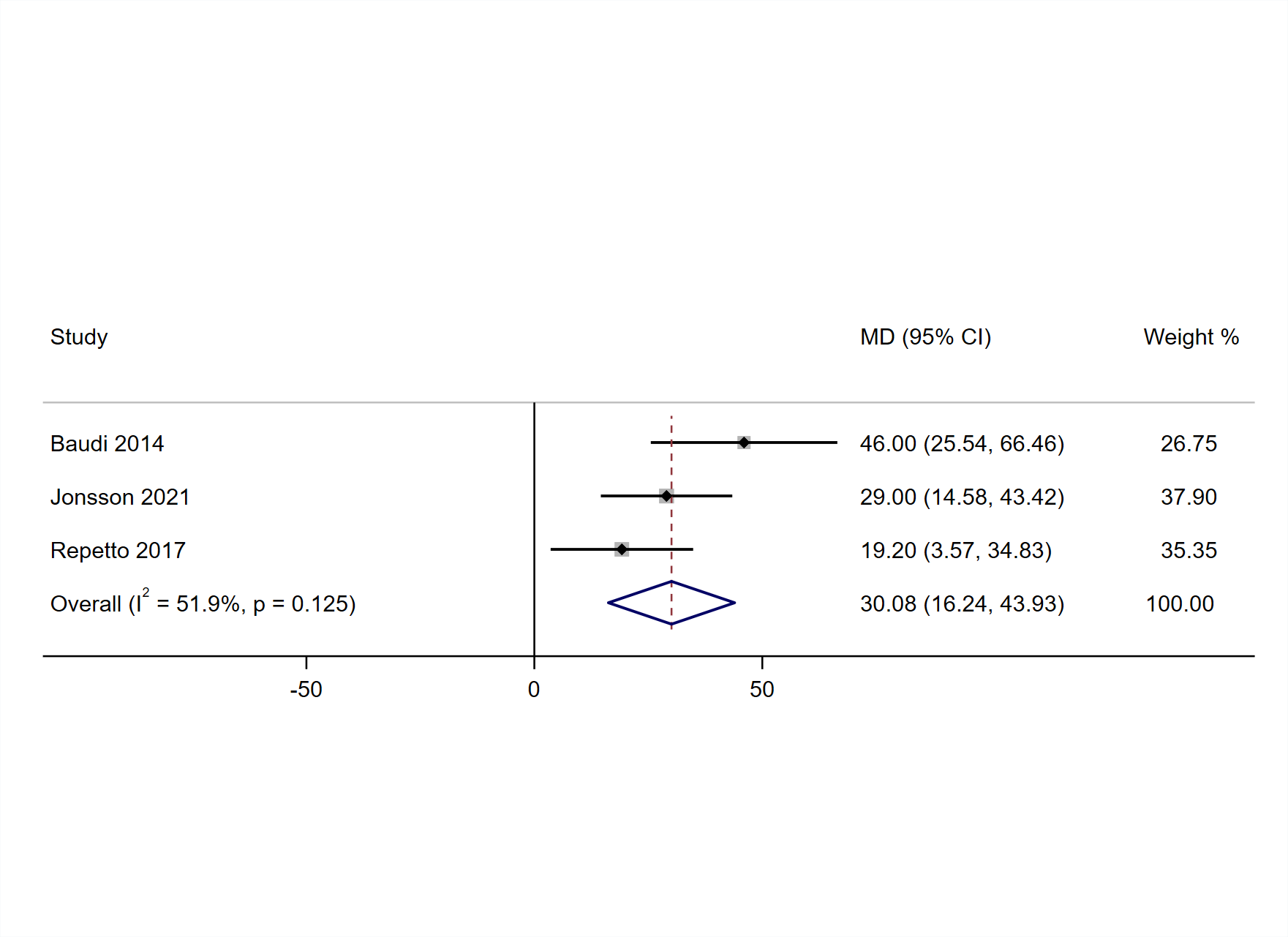

Supplement: Supplementary file 4 — Figure S4. Comparison of postoperative abduction angle between RTSA group and HA group. MD, mean difference; CI, confidence interval. [file OS-17-313-s005.tif]

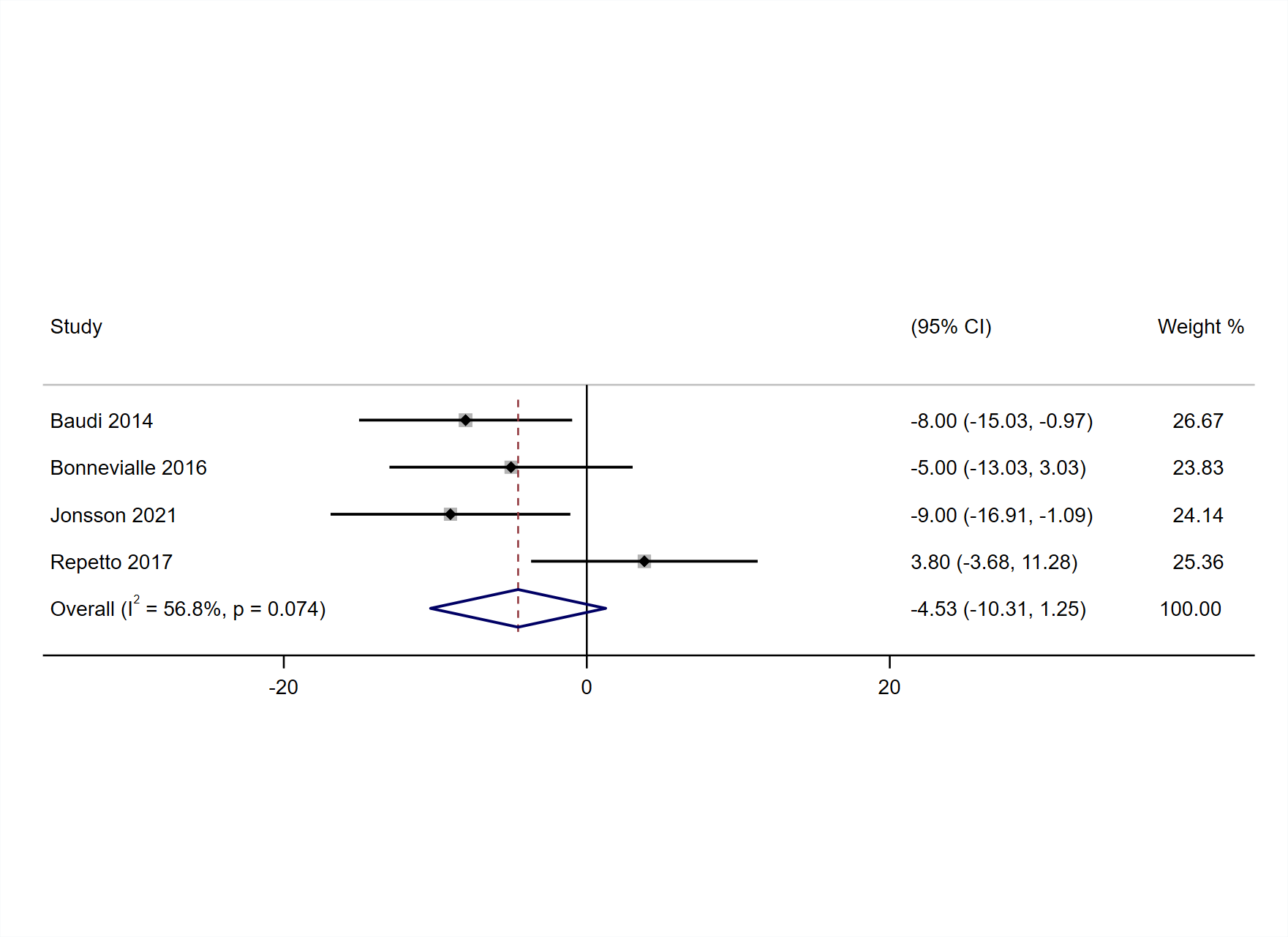

Supplement: Supplementary file 5 — Figure S5. Comparison of postoperative external rotation angle between RTSA group and HA group. MD, mean difference; CI, confidence interval. [file OS-17-313-s014.tif]

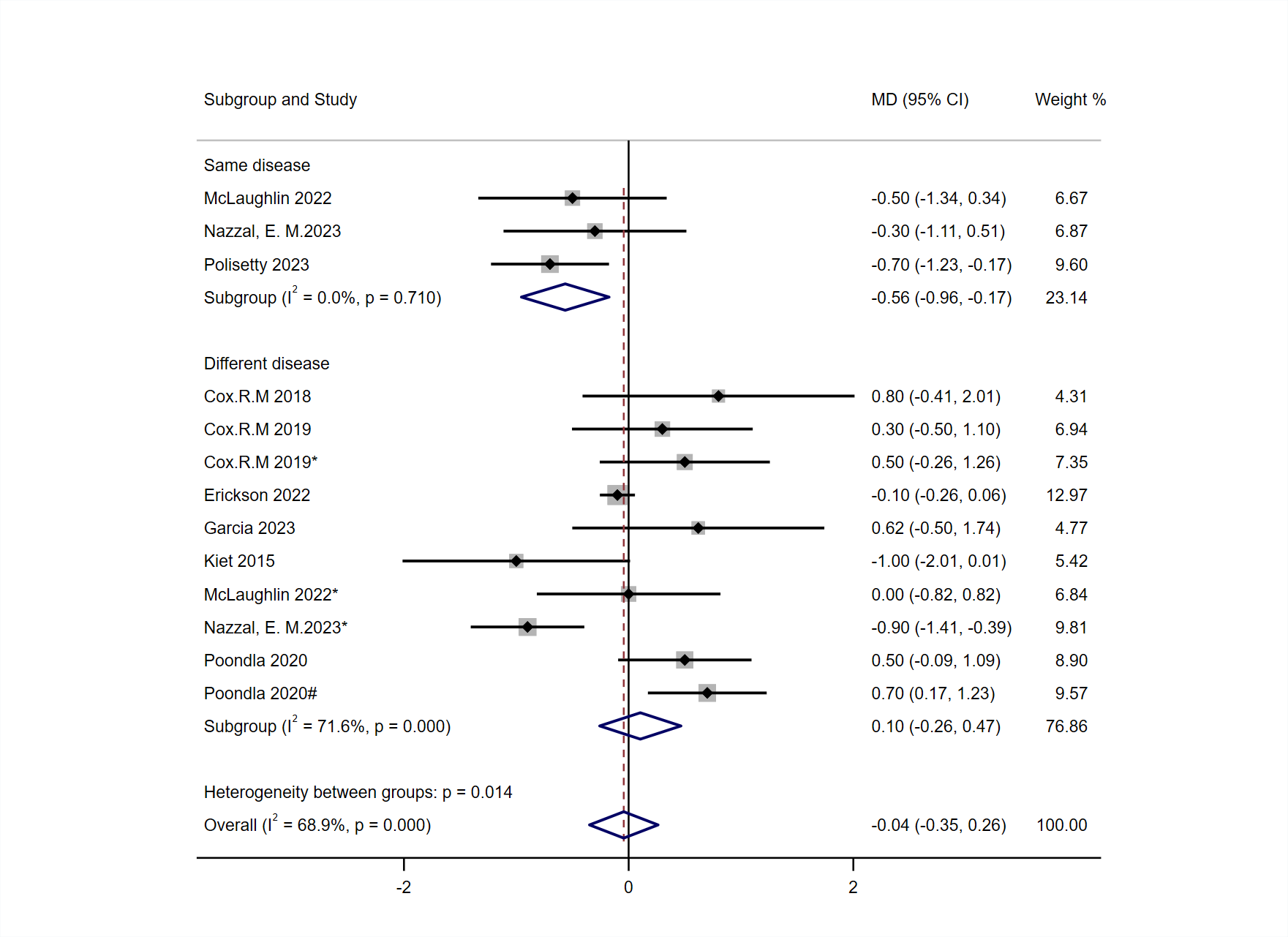

Supplement: Supplementary file 6 — Figure S6. Comparison of postoperative VAS scores between RTSA group and ATSA group. MD, mean difference; CI, confidence interval. [file OS-17-313-s013.tif]

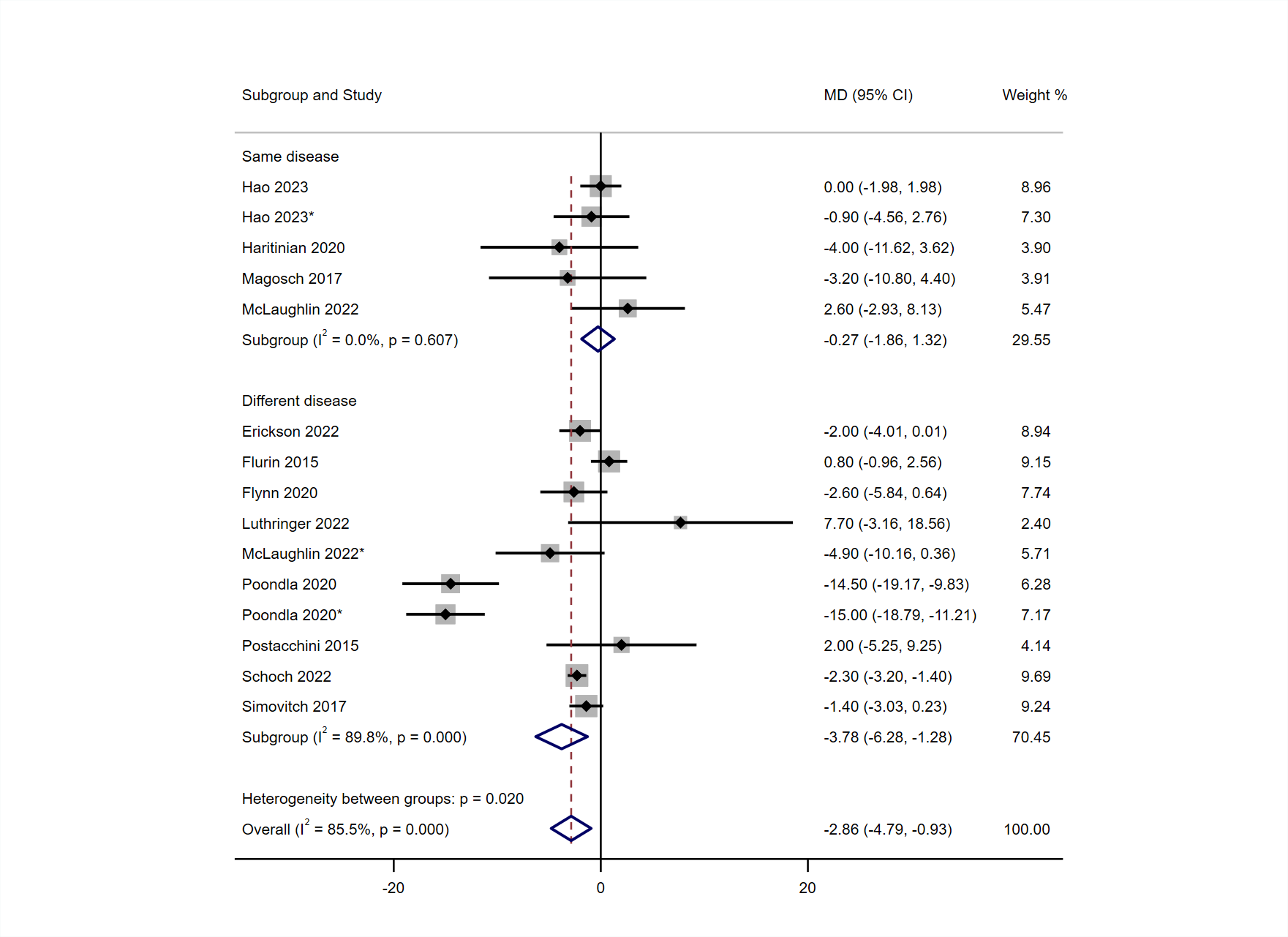

Supplement: Supplementary file 7 — Figure S7. Comparison of postoperative Constant scores between RTSA group and ATSA group. MD, mean difference; CI, confidence interval. [file OS-17-313-s007.tif]

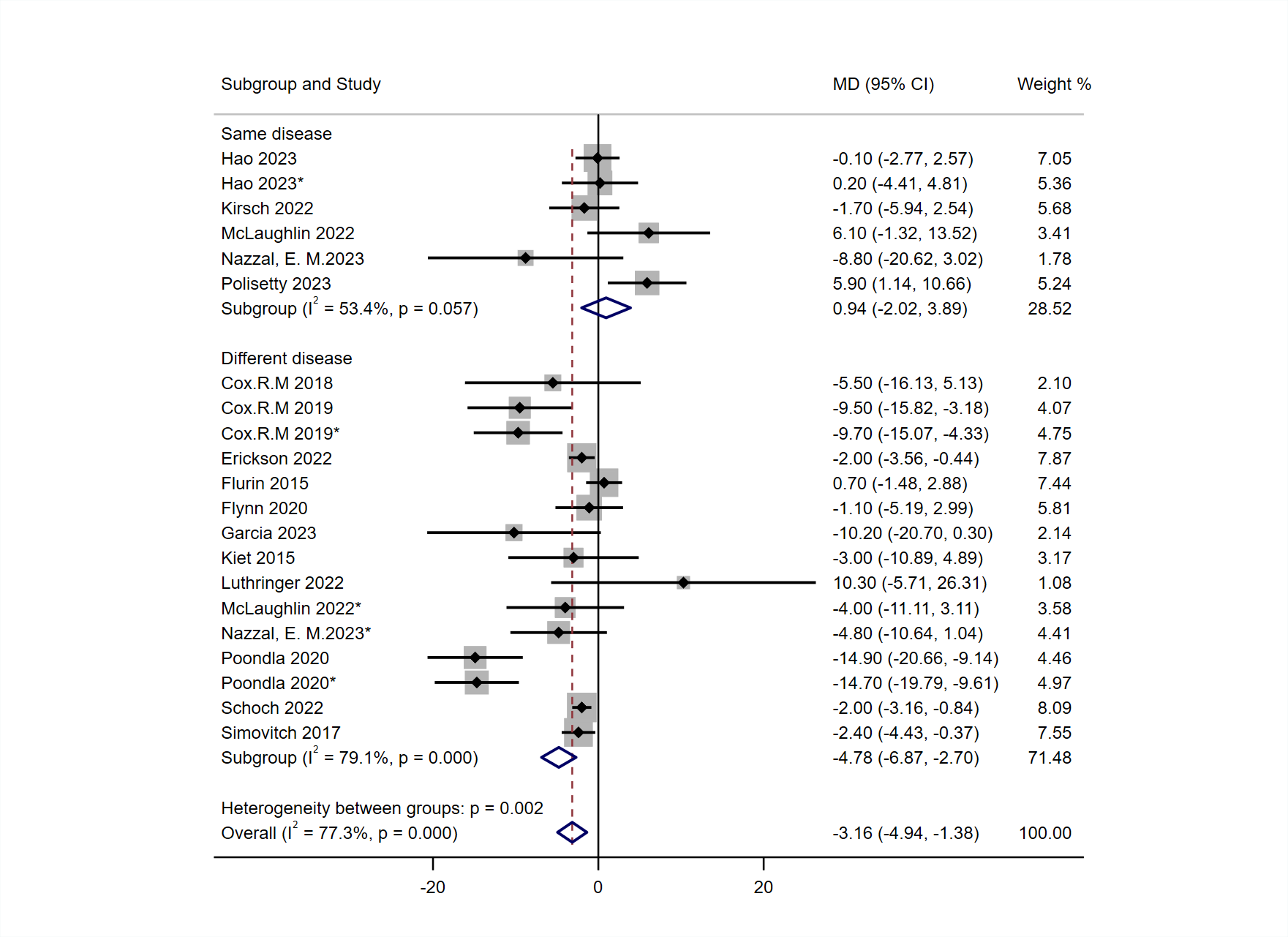

Supplement: Supplementary file 8 — Figure S8. Comparison of postoperative ASES scores between RTSA group and ATSA group. MD, mean difference; CI, confidence interval. [file OS-17-313-s010.tif]

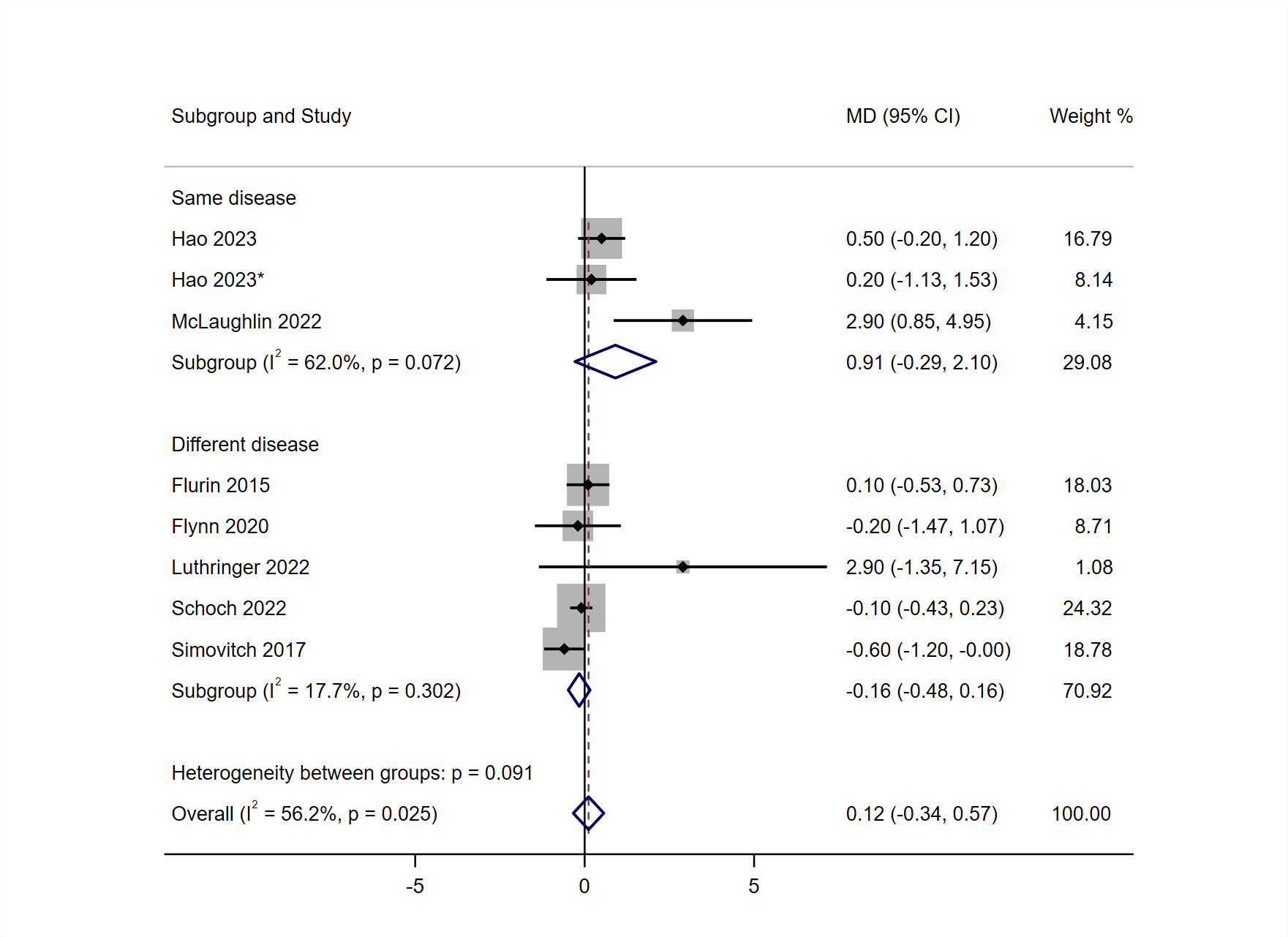

Supplement: Supplementary file 9 — Figure S9. Comparison of postoperative UCLA scores between RTSA group and ATSA group. MD, mean difference; CI, confidence interval. [file OS-17-313-s015.tif]

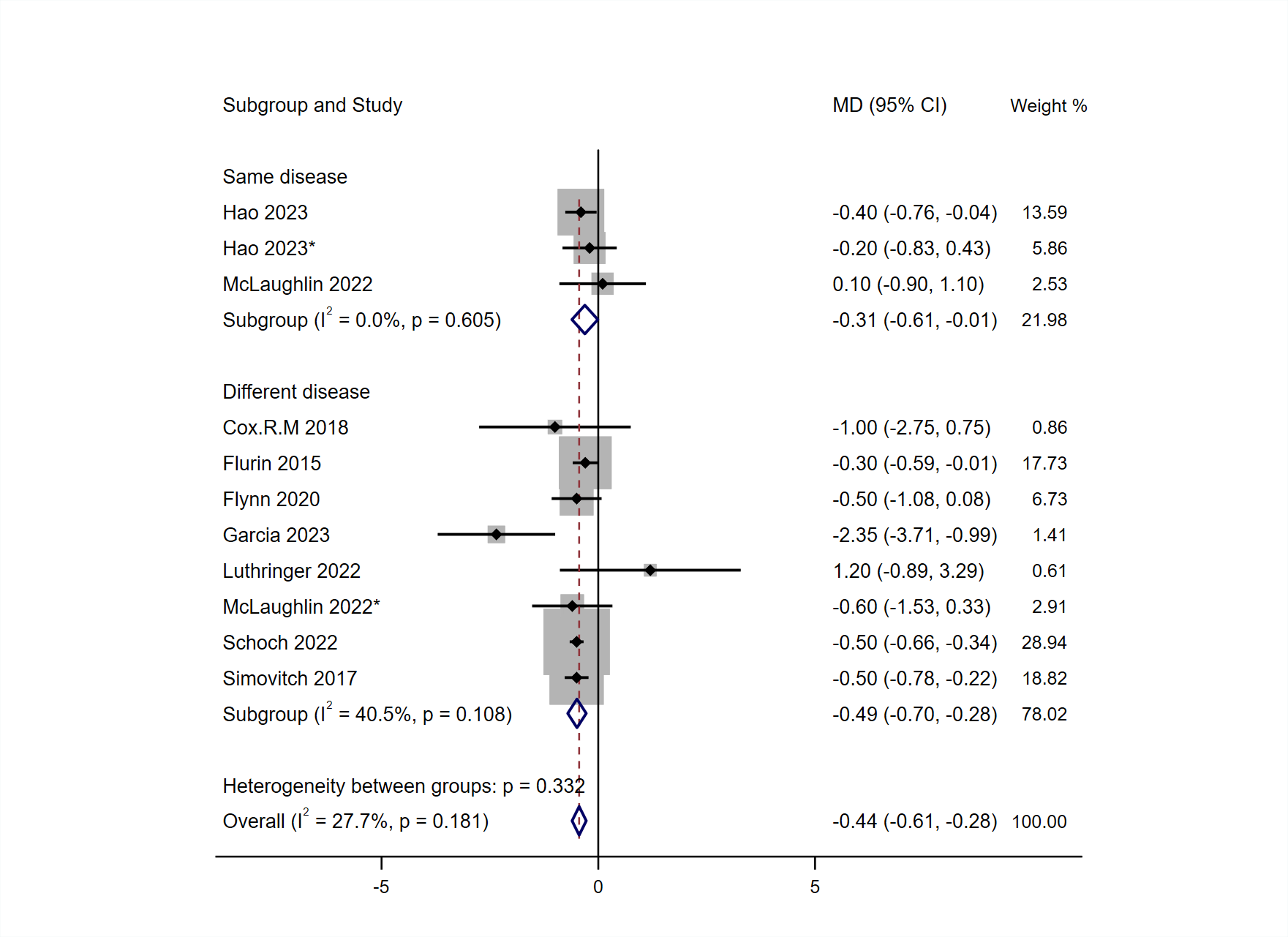

Supplement: Supplementary file 10 — Figure S10. Comparison of postoperative SST scores between RTSA group and ATSA group. MD, mean difference; CI, confidence interval. [file OS-17-313-s006.tif]

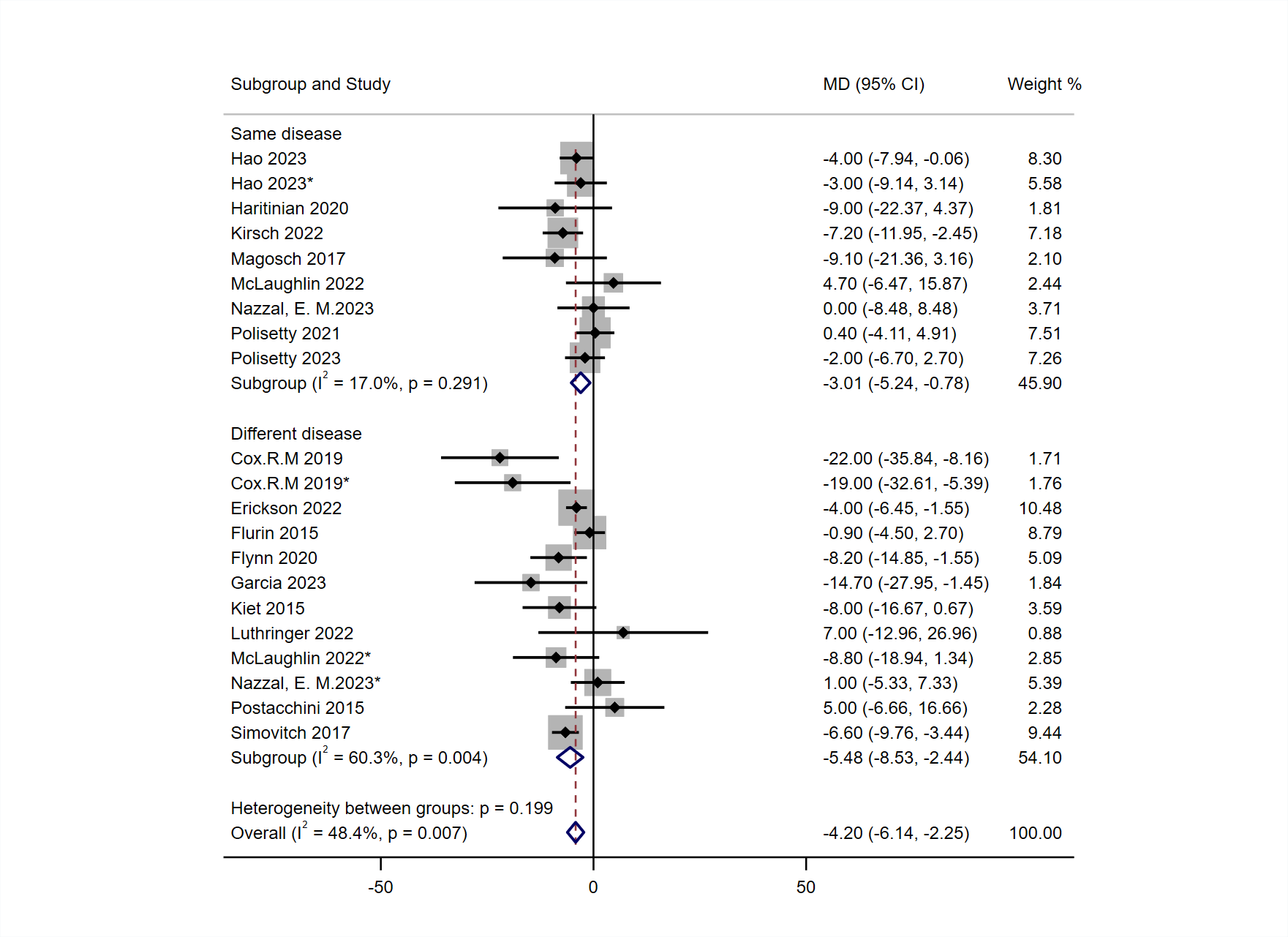

Supplement: Supplementary file 11 — Figure S11. Comparison of postoperative anterior flexion angle between RTSA group and ATSA group. MD, mean difference; CI, confidence interval. [file OS-17-313-s012.tif]

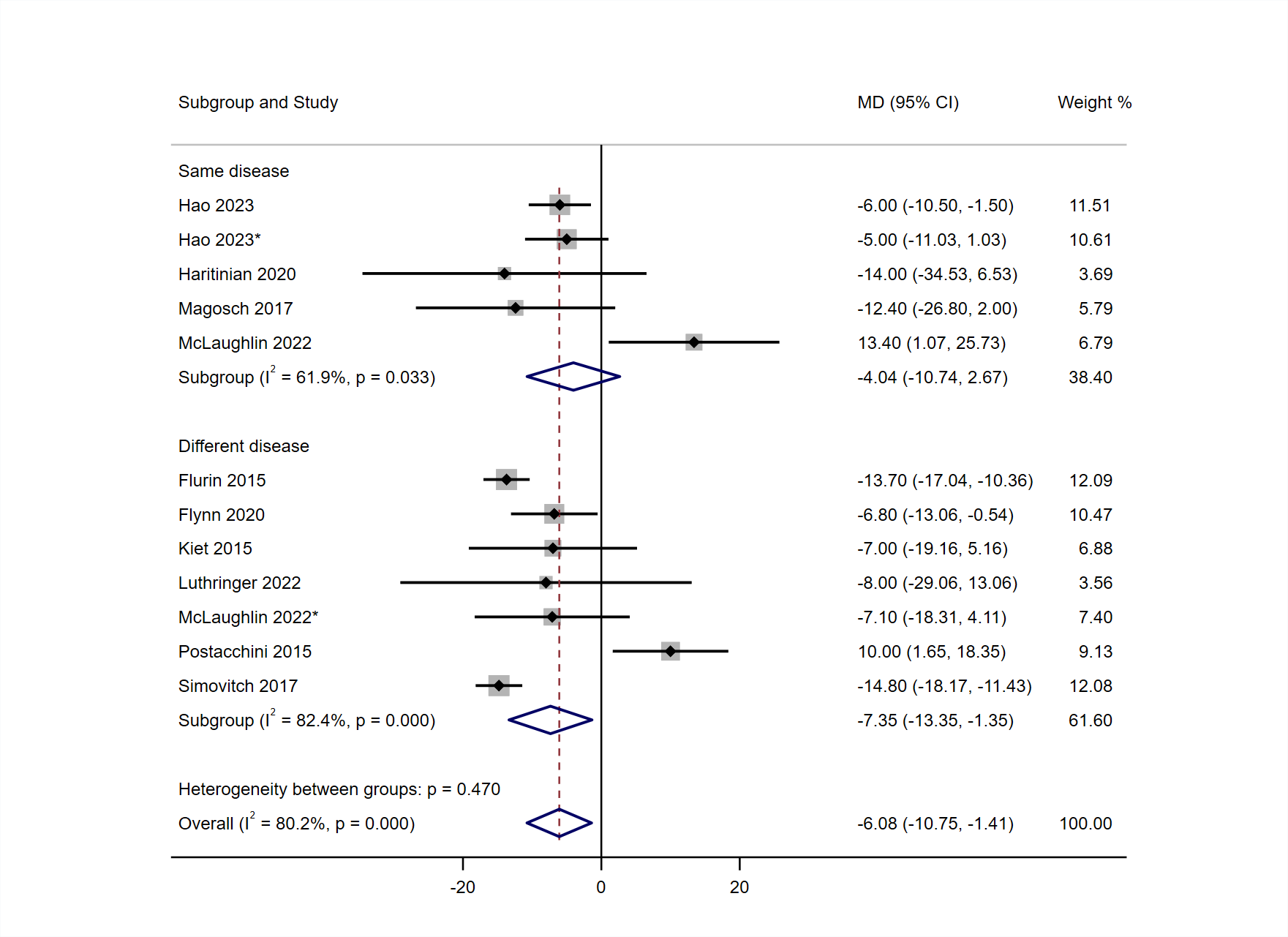

Supplement: Supplementary file 12 — Figure S12. Comparison of postoperative abduction angle between RTSA group and ATSA group. MD, mean difference; CI, confidence interval. [file OS-17-313-s008.tif]

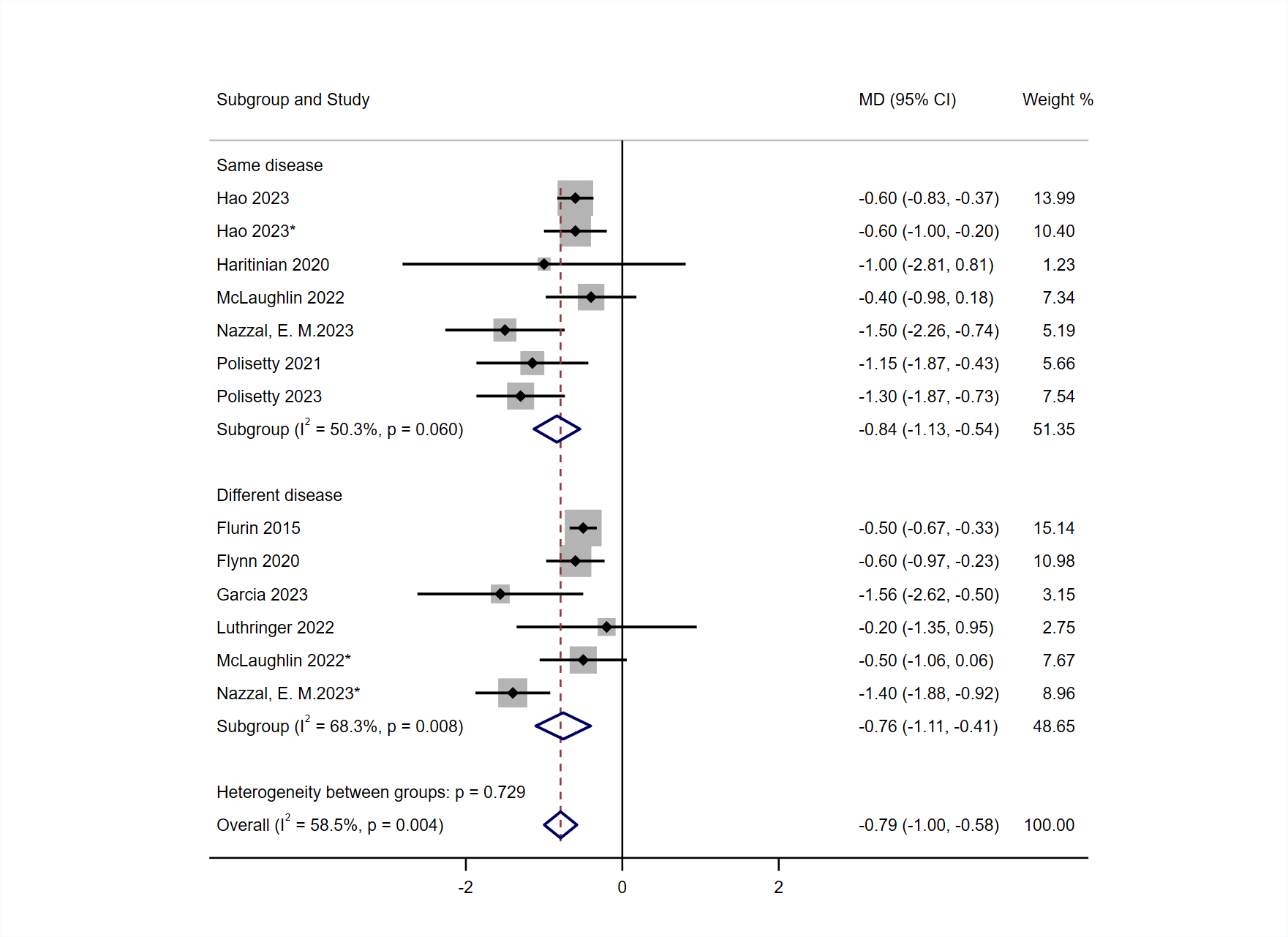

Supplement: Supplementary file 13 — Figure S13. Comparison of postoperative internal rotation angle between RTSA group and ATSA group. MD, mean difference; CI, confidence interval. [file OS-17-313-s001.tif]

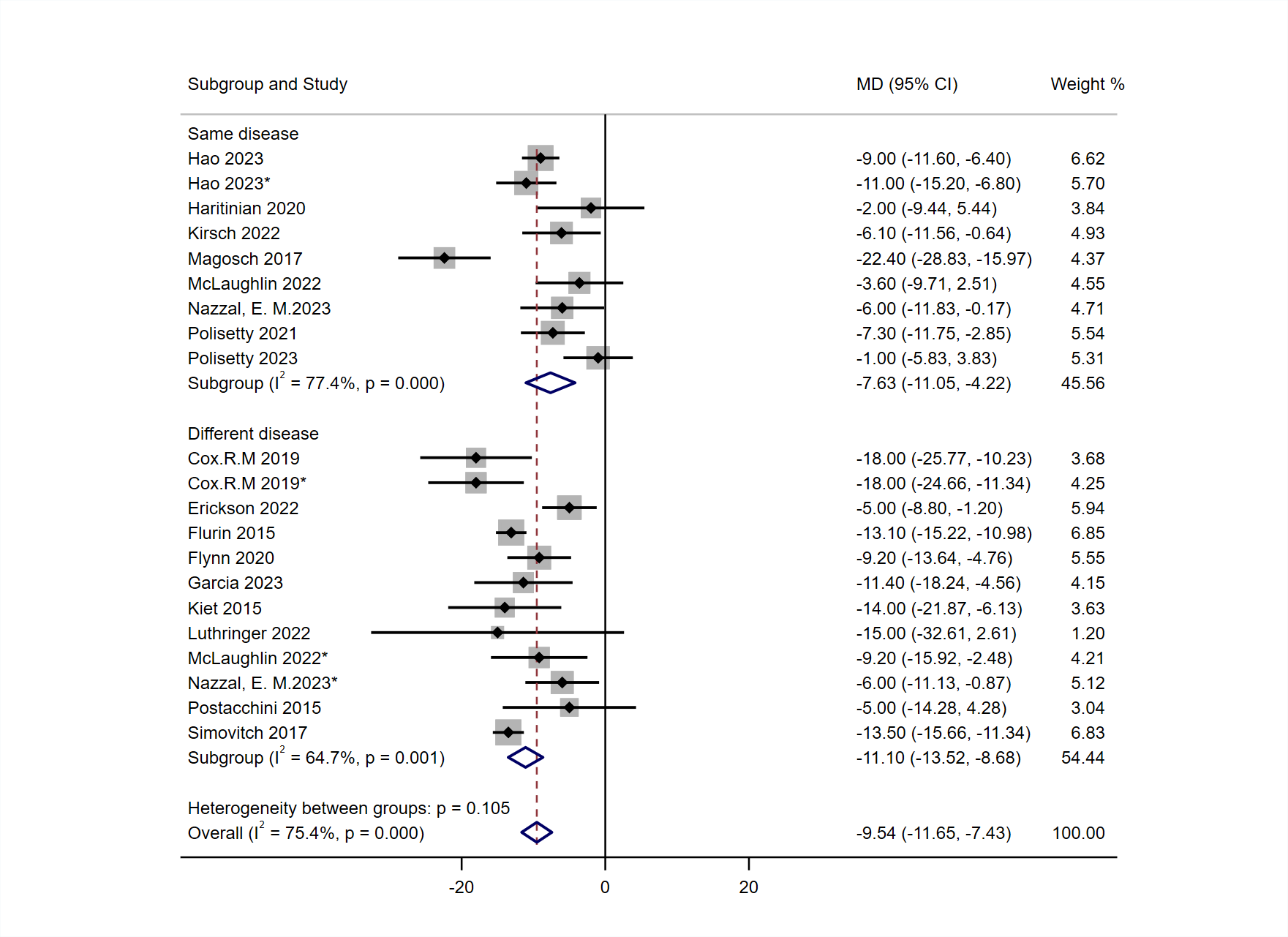

Supplement: Supplementary file 14 — Figure S14. Comparison of postoperative external rotation angle between RTSA group and ATSA group. MD, mean difference; CI, confidence interval. [file OS-17-313-s002.tif]
