# Supplementary material for: Whether the Indications for Reverse Shoulder Arthroplasty Should Continue to Be Expanded? A Systematic Review and Meta‐Analysis
Source: Orthop Surg. 2024 Dec 12;17(2):313–32. doi: 10.1111/os.14311 (PMC11787993; doi:10.1111/os.14311)
Supplement: Supplementary file 15 — Table SI. Postoperative complications and rehabilitation program. Table SII. Summary of outcome measures not included in meta‐analysis (RTSA vs. HA). Table SIII. Summary of outcome measures not included in meta‐analysis (RTSA vs. ATSA). [file OS-17-313-s011.docx]

Table SI：Postoperative complications and rehabilitation program

| Study（surgical of Control） | Complications | |
| --- | --- | --- |
|  | RSA | control |
| ^3^Batar 2020(HA) | infection 2/prosthetic loosening 1/Periprosthesis fracture 1 | infection2/subscapularis definciency 1/dislocation 4/glenoid osteolysis 1 |
| ^4^Baudi 2014(HA) | nerve injury 1 | infection 2/pulmonary embolism1/stiffness 3 |
| ^9^Bonnevialle 2016(HA) | hematoma 1/heterotopic ossification 2/infection 1 | stiffness 11/heterotopic ossification 1/infection 1 |
| ^10^Boyer 2017(HA) | inflammation 5/nerve injury 1/periprosthesis fracture 2/infection 1/osteolysis 4/notches 5/problem with locking 5 | inflammation 5/infection 1/periprosthesis fracture 1/osteolysis 1/problem with locking 2/cuff rupture 3/lymphoedema 1 |
| ^13^Chalmers 2014(HA) | severe pain 1 | arthrofibrosis 1/neuritis 1 |
| ^19^Cuff 2013(HA) | NR | NR |
| ^30^Han 2020(HA) | Glenoid component loosening 6/Implant failure or revision 8/Rotator cuff arthropathy 10/Dislocation 3/Scapular notching 1/Symptoms of nerve stimulation 4 | Glenoid component loosening 2/Implant failure or revision 0/Rotator cuff arthropathy 3/Dislocation 4/Scapular notching 8/Symptoms of nerve stimulation 2 |
| ^35^Jonsson 2021(HA) | NR | NR |
| ^36^Kany 2021(HA and ATSA) | 0 | complex regional pain syndrom 1/Glenoid wear 2;Cuff tear 1/Glenoid wear 3/Instability 1/Deep infection1（ATSA group:pain 1/nerve injury 1/glenoid loosening 4/pulmonary embolism 1） |
| ^39^Kleim 2021(HA and ATSA) | stress fracture 2/hematoma 2/anemia 2/nerve injury 2 | |
| ^42^Laas 2021(HA) | NR | |
| ^45^Leung 2012(HA) | infection 2/instability 1/acromion fractures 2/periprosthetic fracture 1/notches 2/nerve injury 1 | infection 2/continued pain 3 |
| ^64^Repetto 2017(HA) | notches 4/instability 2/ periprosthetic fractures 1/haematoma 1/infection 1) | tuberosities non-union or malunion 5/secondary cuff failure 1/periprosthetic fracture 2/stiffness 2) |
| ^68^Sebastia-Forcada 2014(HA) | hematoma 1/infection 1 | severe pain 6 |
| ^73^Solomon 2016(HA) | dislocation 2 | infection 1 |
| ^77^Yahuaca 2020(HA) | NR | |
| ^2^Aibinder 2019(ATSA) | NR | |
| ^5^Baumgarten 2018(ATSA) | Plate loosening 2/fracture or dislocation 1 | nerve injury 2/arthrofibrosis 2/prosthesis loosening 2 |
| ^17^Cox.R.M 2018(ATSA) | stress fracture 1/deep veinous thrombosis 1 | rotator cuff tear 1/Subscapular muscle repair failed 1/periprosthesis fracture 1 |
| ^16^Cox.R.M 2019(ATSA) | Acromioclavicular joint separation 1/Periprosthesis fracture 1 | hematoma 1/Acromioclavicular joint arthritis 1/clavicle fracture 1 |
| ^21^Erickson 2022(ATSA) | NR | |
| ^23^Flurin 2015(ATSA) | NR | |
| ^24^Flynn 2020(ATSA) | instability 2/Periprosthesis fracture 2/acromion or scapula fracture 3/other 1 | rotator cuff tear 3/prosthesis loosening 4/instability 1/periprosthesis fracture 1/infection 1 |
| ^29^Garcia 2023(ATSA) | acromial fracture 4 | rotator cuff tear 3/prosthesis loosening 2 |
| ^32^Hao 2023(ATSA) | RNC | |
| ^33^Haritinian 2020(ATSA) | NR | |
| ^37^Kiet 2015(ATSA) | infection 2/dislocation 1/fracture 4 | infection 1/rotator cuff injury 4/glenoid loosening 2 |
| ^38^Kirsch 2022(ATSA) | neural paralysis 1/fracture 2 | rotator cuff injury 1/neural paralysis 1/hematoma 1 |
| ^44^Lawrence 2019(ATSA) | NR | |
| ^47^Loew 2023(ATSA) | hematoma 2/infection 6/glenosphere disease 3/prosthesis loosening 2/acromion stress fracture 1/periprosthetic fracture 1 | infection 3/cuff deficiency 2/periprosthetic fracture 1 |
| ^49^Luthringer 2022(ATSA) | infection 2/periprosthetic fracture 1/prosthesis failure 1 | infection 2/glenoid loosening 1 |
| ^50^Magosch 2017(ATSA) | prosthesis loosening 3/infection 2/periprosthetic fracture 1/other 1 | prosthesis loosening 8/dislocation 1/rotator cuff injury 1/glenoid fracture 1 |
| ^51^McLaughlin 2022(ATSA) | acromial fracture 1/scapular fracture 1/dislocation 1 | glenoid loosening 3/pain 2/subscapular muscle tear 1/infection 1 |
| ^52^Merolla 2020(ATSA) | NR | |
| ^53^Mowbary 2022(ATSA) | NR | |
| ^54^Nazzal, E. M.2023(ATSA) | infection 1/fracture 2/dislocation 3 | fracture 4/rotator cuff injury 4 |
| ^55^Parada 2021(ATSA) | acromical and scapular fracture 69/instability 60/pain 49/infection 36/prosthesis loosening 37/humeral fracture 36/nerve injury 15/coracoid and clavicle fracture 7/unclear 63 | rotator cuff injury 34/supscapularis failure 35/prosthesis loosening 63/infection 28/pain 25/nerve injury 15/instability 14/humeral fracture 8/unclear 15 |
| ^60^Polisetty 2021(ATSA) | NR | |
| ^61^Polisetty 2023(ATSA) | acromial fracture/nerve injury/glenoid loosening | infection/subscapular muscle injury/nerve injury |
| ^62^Poondla 2020(ATSA) | infection 1/instability 5/periprosthesis fracture 1 | infection 1/instability 3/Periprosthesis fracture 1/rotator cuff tear 4/prosthesis loosening 5 |
| ^63^Postacchini 2015(ATSA) | infection 3/instability  4/periprosthetic fracture 1 | instability 2/periprosthetic fracture 1/rotator cuff tear 1/rotator cuff tear 1 |
| ^66^Schaller 2022(ATSA) | 0 | dislocation 2/nerve injury 1 |
| ^67^Schoch 2022(ATSA) | NR | |
| ^71^Simovitch 2017(ATSA) | NR | |
| ^74^Triplet 2015(ATSA) | neural paralysis 2/acromion fractures 3 | rotator cuff tear 1 |

Table SII：Summary of outcome measures not included in meta-analysis（RTSA vs.HA）

| **Covariates** | **study** | **RSA(No.)** | **RSA group** | **HA(No.)** | **HA group** | **P value** |
| --- | --- | --- | --- | --- | --- | --- |
| **VAS score** | ^42^Lass 2021 | 17 | 1.0（0.0-1.0） | 14 | 2.5(1.0-4.5) | 0.05 |
|  | ^39^Kleim 2021 | 32 | 1.1 | 21 | 1.57 | NR |
|  | ^13^Chalmers 2014 | 9 | 1±1 | 9 | 3±3 | ＞0.05 |
|  | ^30^Han 2020 | 64 | 3.4±2.3 | 62 | 4.7±2.1 | 0.027 |
| **Constant score** | ^3^Batar 2020 | 33 | 49(13-78) | 25 | 24(9-90) | ＜0.05 |
|  | ^4^Baudi 2014 | 25 | 56.2±14.9 | 28 | 42.3±16.6 | ＜0.05 |
|  | ^10^Boyer 2017 | 65 | 77.6(28.8-119) | 69 | 72(11-120) | NR |
|  | ^35^Jonsson 2021 | 41 | 58.7±16.3 | 43 | 47.7±20 | 0.007 |
|  | ^42^Laas 2021 | 17 | 51(45-68) | 14 | 32(25-57) | ＜0.05 |
|  | ^68^Sebastia-Forcada 2014 | 31 | 56.1(24-80) | 30 | 40(8-74) | 0.001 |
|  | ^36^Kany 2021 HA(CrCo) | 11 | 62(35-83) | 10 | 51(25-80) | NR |
|  | ^36^Kany 2021 HA(PYC) |  |  | 24 | 70(50-80) | NR |
|  | ^39^Kleim 2021 | 32 | 72.9 | 21 | 80.3 | NR |
| **ASES score** | ^3^Batar 2020 | 33 | 70(11.6-88.3) | 25 | 44.6(17.5-98.3) | 0.06 |
|  | ^19^Cuff 2013 | 24 | 77(67-82) | 23 | 62（28-84） | 0.0001 |
|  | ^73^Solomon 2016 | 16 | 79(72-86)‡ | 8 | 59(47-71)‡ | 0.02 |
| **UCLA score** | ^30^Han 2020 | 64 | 24.5±5.5 | 62 | 22.1±7.9 | 0.014 |
|  | ^68^Sebastia-Forcada 2014 | 31 | 29.1(16-34) | 30 | 21.1(6-34) | 0.01 |
| **Forward flexion** | ^3^Batar 2020 | 33 | 120（60-160） | 25 | 70（40-170） | ＜0.05 |
|  | ^10^Boyer 2017 | 65 | 108.7(30-160) | 69 | 99.5(25-160) | NR |
|  | ^19^Cuff 2013 | 24 | 139(102-172) | 23 | 100(30-170) | 0.0002 |
|  | ^42^Laas 2021 | 17 | 110(90-135) | 14 | 90(60-100) | 0.02 |
|  | ^45^Leung 2012 | 36 | 113 | 20 | 58 | ＜0.001 |
|  | ^68^Sebastia-Forcada 2014 | 31 | 120.3(40-180) | 30 | 79.8(20-180) | 0.01 |
|  | ^73^Solomon 2016 | 16 | 110(100-130)‡ | 8 | 104(95-110)‡ | 0.34 |
|  | ^39^Kleim 2021 | 32 | 128 | 21 | 143.1 | NR |
| **Abduction** | ^3^Batar 2020 | 33 | 104(40-180) | 25 | 50(30-160) | ＜0.05 |
|  | ^10^Boyer 2017 | 65 | 99.4(10-150) | 69 | 90.3(35-160) | NR |
|  | ^42^Laas 2021 | 17 | 90(90-110) | 14 | 75(45-80) | 0.01 |
|  | ^68^Sebastia-Forcada 2014 | 31 | 112.9(50-170) | 30 | 78.7(30-150) | 0.001 |
|  | ^39^Kleim 2021 | 32 | 121.7 | 21 | 131.4 | NR |
| **Internal rotation** | ^3^Batar 2020 | 33 | 40(10-80)† | 25 | 50(0-90)† | 0.19 |
|  | ^4^Baudi 2014 | 25 | 3.3 | 28 | 3 | ＞0.05 |
|  | ^9^Bonnevialle 2016 | 41 | Scacrum | 57 | L3 | 0.03 |
|  | ^19^Cuff 2013 | 24 | 46% | 23 | 30% | 0.27 |
|  | ^42^Laas 2021 | 17 | 40(25-54) | 14 | 40(30-58) | 0.94 |
|  | ^45^Leung 2012 | 36 | T12 | 20 | L3 | 0.397 |
|  | ^64^Repetto 2017 | 27 | gluteus level | 24 | lombosacral junction | ＞0.05 |
|  | ^39^Kleim 2021 | 32 | 2.3 | 21 | 2.7 | NR |
| **External rotation** | ^3^Batar 2020 | 33 | 50(0-90)† | 25 | 30(0-90)† | 0.21 |
|  | ^10^Boyer 2017 | 65 | 20.9(10-80) | 69 | 28.3(0-55) | NR |
|  | ^19^Cuff 2013 | 24 | 24(8-42) | 23 | 25(0-48) | 0.88 |
|  | ^42^Laas 2021 | 17 | 20(10-60) | 14 | 30(20-40) | 0.44 |
|  | ^39^Kleim 2021 | 32 | 36.3 | 21 | 44.3 | NR |
|  | ^45^Leung 2012 | 36 | 33 | 20 | 30 | 0.672 |
|  | ^73^Solomon 2016 | 16 | 28(25-30)‡ | 8 | 29(22-35) | 0.8(at 90°) |

†:interquartile range; ‡:95% CI

Table SIII：Summary of outcome measures not included in meta-analysis（RTSA vs.ATSA）

| **Covariates** | **study** | **RSA(No.)** | **RSA score** | **ATSA(No.)** | **ATSA score** | **P value** |
| --- | --- | --- | --- | --- | --- | --- |
| **Constant score** | ^36^Kany 2021 TSA | 11 | 62(35-83) | 77 | 67(26-97) | NR |
|  | ^39^Kleim 2021 | 32 | 72.9 | 23 | 84 | NR |
|  | ^49^Luthringer 2022 | 83 | 59.9 | 17 | 55.4 | 0.402 |
|  | ^52^Merolla 2020 | 32 | 68(66-76.5)† | 26 | 80（75-82）† | ＜0.001 |
| **ASES score** | ^5^Baumgarten 2018 | 42 | 82(30-100)† | 80 | 87(15-100)† | NR |
|  | ^44^Lawrence 2019 | 41 | 83.3† | 50 | 95.0† | ＜0.001 |
|  | ^49^Luthringer 2022 | 83 | 74 | 17 | 68.8 | ＜0.01 |
|  | ^74^Triplet 2015 | 33 | 82(53-93)† | 18 | 91(78-95)† | 0.035 |
| **SST score** | ^5^Baumgarten 2018 | 42 | 7(0-12) | 80 | 9(0-12) | NR |
|  | ^49^Luthringer 2022 | 83 | 8.7 | 17 | 7.8 | ＜0.01 |
|  | ^74^Triplet 2015 | 33 | 9(5-11) | 18 | 9(7-12) | 0.137 |
| **Forward flexion** | ^39^Kleim 2021 | 32 | 128 | 23 | 144 | NR |
|  | ^2^Aibinder 2019 | 65 | 129（30-170） | 35 | 129.7（20-170） | NR |
|  | ^49^Luthringer 2022 | 83 | 123 | 17 | 114 | 0.437 |
|  | ^52^Merolla 2020 | 32 | 160(150-170)† | 26 | 170(160-170)† | 0.072 |
|  | ^66^Schaller 2022 | 31 | 112 | 44 | 128 | ＜0.0001 |
|  | ^74^Triplet 2015 | 33 | 125(100-135) | 18 | 150(120-155) | 0.003 |
| **Abduction** | ^21^Erickson 2022 | 154 | 10.1±4.7 | 155 | 10±4.4 | 0.729（Strength in lbs） |
|  | ^39^Kleim 2021 | 32 | 121.7 | 23 | 138.3 | NR |
|  | ^49^Luthringer 2022 | 83 | 102 | 17 | 102 | 0.906 |
|  | ^52^Merolla 2020 | 32 | 150(150-160)† | 26 | 160(150-170)† | 0.006 |
|  | ^66^Schaller 2022 | 31 | 103 | 44 | 122 | ＜0.0001 |
|  | ^74^Triplet 2015 | 33 | 90(75-105)† | 18 | 90(84-101)† | 0.488 |
| **Internal rotation** | ^2^Aibinder 2019 | 65 | sacrum to L4 | 35 | sacrum to L4 | NR |
|  | ^16^Cox 2019 | 13 | L4 | 26 | L1 | 0.045 |
|  | ^16^Cox 2019# | 13 | L2 | 26 | L1 | 0.177 |
|  | ^21^Erickson 2022 | 154 | 40±18 | 155 | 40±18 | 0.902（at 90°） |
|  | ^37^Kiet 2015 | 53 | L2±4 | 47 | L2±3 | 0.872 |
|  | ^39^Kleim 2021 | 32 | 2.3 | 23 | 3.1 | NR |
|  | ^49^Luthringer 2022 | 83 | 4.2 | 17 | 4 | 0.667 |
|  | ^52^Merolla 2020 | 32 | 8(5-8)† | 26 | 6(6-8)† | 0.854 |
|  | ^63^Postacchini 2015 | 12 | D8 (L3–D6) | 12 | D8 (L2–D6) | NR |
| **External rotation** | ^2^Aibinder 2019 | 65 | 40.3(0-90) | 35 | 43.4(5-90) | NR |
|  | ^39^Kleim 2021 | 32 | 36.3 | 23 | 40 | NR |
|  | ^49^Luthringer 2022 | 83 | 28 | 17 | 33 | 0.419 |
|  | ^52^Merolla 2020 | 32 | 20(12.5-30)† | 26 | 22.5(15,45)† | 0.269 |
|  | ^66^Schaller 2022 | 34 | 26 | 44 | 40 | ＜0.001 |
|  | ^74^Triplet 2015 | 33 | 40(25-48)† | 18 | 45(40-60)† | 0.005 |

†:interquartile range; ‡:95% CI
